# Destabilization of Structured RNAs by OPC and TIP4PD Water Models

**DOI:** 10.1021/acs.jctc.5c01678

**Published:** 2026-02-10

**Authors:** Miroslav Krepl, Vojtěch Mlýnský, Agnesa Rusnáková, Pavel Banáš, Michal Otyepka, Jiří Šponer

**Affiliations:** † 86853 Institute of Biophysics of the Czech Academy of Sciences, Královopolská 135, 612 00 Brno, Czech Republic; ‡ Czech Advanced Technology and Research Institute, CATRIN, 48207 Palacký University, Křížkovského 511/8, Olomouc 779 00, Czech Republic; § IT4Innovations, VSB-Technical University of Ostrava, 17. listopadu 2172/15, 708 00 Ostrava-Poruba, Czech Republic

## Abstract

The four-point OPC water model has recently gained a reputation as the preferred choice for molecular dynamics (MD) simulations of nucleic acids and proteins, providing more realistic reproduction of bulk physical properties of water than the older three-point models. It has been shown to improve, for example, simulations of unstructured biomolecules such as RNA tetranucleotides or intrinsically disordered proteins. However, the performance for folded RNA structures was not specifically explored. Here we present extensive testing of the OPC water model on three different RNAs with intricate tertiary structures – the ribosomal L1 stalk RNA-protein protuberance, the mini tetraloop-tetraloop receptor (miniTTR-6) folded RNA, and the GAAA tetraloop-tetraloop receptor homodimer. The OPC performance is directly compared with SPC/E, TIP3P, and OPC3 water models using the OL3 AMBER RNA force field (FF). We found substantial effect of the water model on simulation behavior. For all three systems, we observe large-scale unfolding of the RNA, and even loss of the L1 stalk protein–RNA interface, when simulated with the OPC. In contrast, the simulations are entirely stable with the three-point water models. The underlying cause seems to be the higher affinity of the OPC waters to H-bond donor and acceptor groups of the RNA, which weakens the native solute–solute interactions. An identical issue is observed also for the similar and widely used TIP4PD water model combined with the DES-Amber RNA FF. Importantly, the structural consequences of these issues may range from significant structural perturbations to minimal or undetectable effects, depending on the RNA system. Accordingly, we do not claim that the imbalance in water–RNA interactions identified here is a general feature of all RNA molecules. Indeed, simulations of three additional, less structurally complex RNA systems revealed more balanced performance, with no observable differences between water models for the noncanonical 5S rRNA Loop E double helix. However, certain caution is warranted when using the four-point OPC and TIP4PD water models for simulations of structured RNAs, particularly those rich in 2′-OH hydroxyl-based tertiary interactions. For at least some structured RNA systems, the three-point water models may provide more stable behavior in combination with current AMBER RNA force fields.

## Graphical abstract

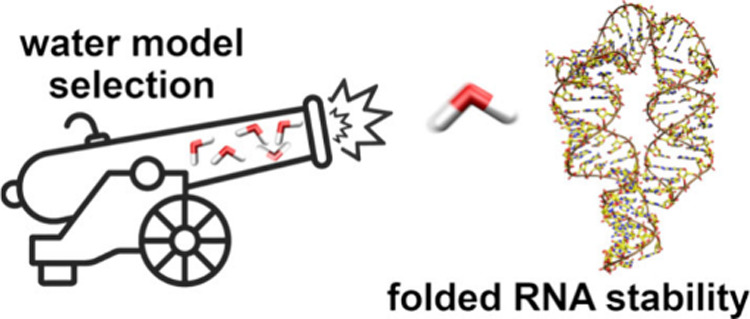

## Introduction

Due to their nature as a charged biopolymer, the nucleic acids can be sensitive to a water model chosen in explicit-solvent molecular dynamics (MD) simulations.^1–16^ An important part of the parametrization efforts aimed at improving the force-field (FF) performance for RNA have therefore been identifying the best performing water model.^2,17,18^ The four-point OPC water model^19^ has recently come to be regarded as the optimal choice for nucleic acid MD simulations. When combined with RNA phosphate parameters refined by Steinbrecher et al.^20^ and the OL3 AMBER RNA FF,^21^ the use of OPC improves the description of single-stranded RNA molecules, such as tetranucleotides (TNs).^2^ This result has been confirmed in subsequent studies investigating RNA single-strands and tetraloops (TLs).^17,18,22–27^ Many recent attempts to improve the AMBER RNA FF or to parametrize a new nucleic acid FF from the scratch were also made with the OPC water model.^22,25,26,28,29^ In addition, the AMBER ff19SB protein FF^30^ recommends the use of the OPC as well, due to its improved performance for intrinsically disordered proteins.^31^ However, beyond the TNs, TLs, and A-RNA duplexes, the OPC water model remains rather untested on more complex RNA systems, despite RNA’s ability to form highly intricate folded structures.^32^

In this work, we assess the performance of the four-point OPC water model on three different structured RNA systems and compare it with other water models such as TIP3P,^33^ SPC/E,^34^ OPC3,^35^ and TIP4PEW.^36^ The common OL3 RNA FF is used to describe the RNA in these simulations. We also evaluate another commonly used four-point water model, the TIP4PD,^37^ in combination with the DES-Amber RNA FF.^38^ Note that the design of four-point water models differs from that of three-point models by the presence of an additional off-atom charge site, intended to provide a more realistic electrostatic potential and, consequently, a more accurate representation of bulk water properties.

The first tested system, the *L1 stalk*, is a flexible protuberance of the large ribosomal subunit, playing a crucial role in protein synthesis.^39^ It facilitates the release of deacylated tRNA after amino acid transfer and ensures smooth elongation cycle progression.^40–43^ Its apex rRNA structure (hereafter referred to as the L1 stalk rRNA) has a highly conserved and autonomously folding structure that is rich in noncanonical RNA interactions, including noncanonical base pairs and triplets, and base–phosphate, base–sugar, and sugar–phosphate interactions.^44,45^ It contains several recurrent RNA motifs, including two kink-turns^46^ and a TL.^47^ We have simulated the L1 stalk rRNA with as well as without the bound L1 protein.

The second tested system, the *mini tetraloop–tetraloop receptor 6* (miniTTR-6) structure, was specifically designed as a very stable structural element for RNA nanotechnology applications.^48^ While the miniTTR-6 lacks the biological significance of the ribosomal L1 stalk, the fact it has been deliberately engineered for high stability^48^ makes it a useful benchmark system as basically any reliable FF should maintain its structure over affordable time scales of standard MD simulations without major structural changes. The tetraloop–tetraloop receptor (TTR) interaction is the key long-range tertiary contact in the system.^49^

The last of the tested systems is a simple symmetric homodimeric system consisting of two RNA duplexes interacting with each other via two equivalent TTR motifs.^50^ These two TTR motifs are the same as in the miniTTR-6, and the system will be henceforth referred to as *hTTR* (homodimeric TTR).^51^ The hTTR was chosen as a testing system as its folding does not require the magnesium ions;^51^ in fact, the isolated TTR forms in complete absence of magnesium and in the presence of a moderate excess of KCl.^49^ There is experimental evidence that even the miniTTR-6 system may not require the magnesium to fold in a sufficient excess of monovalent cations.^52^ All the studied systems are visualized in Supporting Information, Figure S1. In addition to these three primary RNA systems, we also examined a set of other RNA structures to assess whether the observed behavior can be generalized (Supporting Information).

Our standard simulations reveal that the OPC and TIP4PD water models destabilize key tertiary RNA–RNA interactions in all three systems compared to three-point water models. In many cases, this leads to visually striking structural changes of the RNA on unrealistically short time scales. For the L1 stalk, we even observe collapse of the protein-RNA interface that occurs independently of the RNA fold disruptions. The structural changes observed with the OL3/OPC combination are sometimes reversible on the simulation time scale whereas the DES-Amber/TIP4PD leads to more permanent disruptions. However, qualitatively identical structural changes are observed with both FF and water model combinations.

Based on detailed analysis of all systems and additional model calculations directly comparing the OPC and TIP4PD water models to SPC/E, we propose the imbalance introduced by these four-point water models lies in their increased affinity to RNA H-bond donor and acceptor groups, disrupting the native RNA–RNA interactions. Though the difference is subtle for the individual H-bonds (∼0.1–0.3 kcal/mol) and therefore likely negligible for small systems, it may accumulate with the numerous interactions present in large structured RNAs and collectively determining the stability. For large folded RNA systems, this is apparently enough to significantly shift the free energy minima away from the native RNA fold toward more extended and therefore more solvated structures. In principle, all solute–solute H-bonds are affected by this. However, the tertiary interactions formed by the RNA 2′-OH groups are possibly the most sensitive and quickest to respond due to the complex balance between direct and water-mediated interactions. These interactions are omnipresent in large folded RNAs and include, e.g., the A-minor interaction^53^ – the most common tertiary RNA–RNA interaction in large RNAs.

In summary, we suggest that three-point water models (e.g., TIP3P, SPC/E, and OPC3) may be a more reliable choice for simulations of certain structured RNAs with intricate tertiary interactions than four-point water models. The tested three-point water models can be regarded as equivalent in their lack of any tendency to destabilize the native RNA folds. There may still be more subtle system-specific differences that make one of these models more suitable for particular systems. Similarly, there will undoubtedly be RNA systems for which even the four-point water models perform well and do not lead to destabilization of the RNA fold, as also demonstrated here in a subset of our simulations (Supporting Information). In other words, we do not claim that the imbalance reported here applies universally to all RNA molecules. However, somewhat increased caution is warranted when using the four-point OPC and TIP4PD water models, especially in simulations of RNAs that extensively rely on 2′-OH-based tertiary interactions.

## Methods

### Starting Structures

We used the X-ray structure of the L1 stalk rRNA in complex with the L1 protein from *T. thermophilus* (PDB: 3U4M)^44^ as the starting structure for our simulations of the L1 stalk protein–RNA complex. Simulations of isolated L1 stalk rRNA were performed by removing the protein atoms. The cytidine 2111 was always N3-protonated in the starting structure. For simulations of the isolated L1 stalk rRNA, we also utilized its X-ray structure from *H. marismortui* (PDB: 5ML7).^54^ For the simulations of the miniTTR-6, we used its X-ray structure (PDB: 6DVK).^48^ Lastly, the first ensemble frame of the NMR structure of hTTR complex (PDB: 2I7Z) was used as the starting structure for its simulations. To obtain further insights, during the revision we have performed simulations on some additional systems, namely 5S rRNA Loop E noncanonical duplex, the Sarcin–Ricin loop, and an RNA three-way junction. The details of these simulations are provided in the Supporting Information.

All experimentally determined water molecules and monovalent ions were kept in the starting structures. Any experimentally detected monovalent ions other than K^+^ and Cl^–^ were converted into the latter ions. All five experimentally determined X-ray magnesium ions were included in selected simulations of the miniTTR6 system. The crystallization solution of the miniTTR6 also contained 2 mM cobalt hexamine in addition to 20 mM MgCl_2_, resulting in four divalent cobalt ions resolved in the structure.^48^ These cobalt ions were converted to magnesium during system preparation, giving nine bound Mg^2+^ ions in the starting structure of miniTTR6. None of the cobalt ions participated in bridging tertiary interactions.

Besides simulations of folded RNAs, we have also performed simulations of the isolated nucleosides, with their structures taken from the AMBER residue library.

### Force-Field Selection

All coordinates and topology files were generated using the xLeap module of AMBER 22.^55^ The RNA systems were parametrized with the ff99bsc0χ_OL3_ (OL3) FF,^21,56,57^ which is currently the recommended first-choice AMBER FF for RNA.^58^ In a few simulations, the OL3 FF was used with the adjusted phosphate van der Waals (vdW) parameters by Steinbrecher et al.;^20^ this version is abbreviated as OL3_CP_ (CP stands for “Case phosphate”).^20,21^ Note that the OL3_CP_ FF is also sometimes labeled as “LJbb” FF, but it is in fact only a minor modification of the basic OL3 FF. The reason for its testing was that the inclusion of adjusted phosphate vdW parameters together with the OPC water model was reported to somewhat improve the simulations of RNA TNs.^2^ The present simulations indicate that the CP modification does not have any effect on the instabilities observed for the folded RNAs tested here. For the protein component in the simulations of the L1 stalk protein–RNA complex, we used either the ff14SB^59^ or the newer ff19SB^30^ protein FF. The ff19SB FF was directly parametrized to be used with the OPC water model.^30^ To test the TIP4PD water model, we have used the DES-Amber FF for the RNA^38^ and the protein.^60^

### System Building

All simulated systems except the DES-Amber and nucleoside model simulations (see below) were first solvated in an octahedral box filled with water molecules, ensuring a minimum of 12 Å between any solute atom and the box edge. Unless specified otherwise, an excess KCl salt concentration of 0.15 M was achieved by randomly placing KCl ions around the solute; see below a separate paragraph detailing all the water models and ion parameters we tested. Energy minimization and equilibration procedures were performed with pmemd.MPI in AMBER 22, following the established protocol.^61^ Production simulations were conducted using pmemd.cuda^62^ on RTX 3080ti GPUs, with a typical length ranging between 3 to 10 μs. In all cases, multiple independent simulations were initiated with independent equilibration procedures and different random seeds for initial atomic velocities to generate statistically robust ensembles. SHAKE^63^ constraints and hydrogen mass repartitioning^64^ were applied in all simulations unless specified otherwise, enabling a 4 fs integration time step. Long-range electrostatics were treated with the particle mesh Ewald method^65^ under periodic boundary conditions, with a nonbonded LJ interaction cutoff of 9 Å. Temperature and pressure were maintained using a Langevin thermostat and Monte Carlo barostat, respectively. Because DES-Amber parameters are not currently implemented in AMBER, simulations using the DES-Amber RNA FF combined with the TIP4PD water model were performed in GROMACS2020.^66^ The simulation protocol in GROMACS2020 slightly differed from the one in AMBER 22 due to differences in the simulation codes. Specifically, GROMACS simulations were performed in a rhombic dodecahedral box and covalent bonds involving hydrogens were constrained using the LINCS algorithm.^67^ The cutoff distance for the direct space summation of the electrostatic interactions was 12 Å and the simulations were performed using the stochastic velocity rescale thermostat^68^ and Parrinello–Rahman barostat.^69^

### Water Models and Ion Parameters

For most of the water models tested in this work – TIP3P,^33^ SPC/E,^34^ OPC3,^35^ TIP4PEW^36^ and OPC^19^ – there are multiple possible choices of compatible ion parameters available and their use is not standardized in the literature. We have finally decided to chiefly utilize the combinations currently present in the latest default AMBER source packages of Leap^58^ for each water model (i.e., leaprc.water.*; where * is the water model), as we expect this is how the majority of people will be performing their MD simulations. This gave us the standard combinations of Joung–Cheatham (JC) KCl parameters^70^ to be used with the TIP3P, SPC/E and TIP4PEW water models and Li–Merz (LM) parameters^71,72^ for the OPC and OPC3 water models. Nevertheless, limited cross-testing of these parameters was also carried out for selected systems to verify the potential influence of the ion parameters on the observed structural transitions. The simulations strongly suggest that the choice of monovalent ion parameters does not have any effect on the properties investigated in the present study. For the magnesium ions, the 6–12 Li–Merz parameters^73^ were utilized in all cases. Because of computational demands, not all water models were evaluated for every system. In such cases, SPC/E was used as the default representative three-point water model. Based on the results presented here and on prior study,^74^ TIP3P, another widely used three-point water model, is expected to exhibit performance comparable to SPC/E with respect to the RNA fold stabilization examined in this work. The OPC3 model is not widely used in the literature but has, for example, been utilized in a recent FF development study.^75^ DES-Amber simulations with the TIP4PD water model were always run with the scaled CHARMM22 ^76^ ion parameters.^38^

### REST2 Enhanced Sampling Simulations of the hTTR System

Compared to the other systems, the hTTR showed slower conformational developments (see below) in standard simulations. Thus, for this system, we have also performed REST2 ^77^ enhanced sampling simulations. The number of replicas for the calculations done in AMBER was 7, with the scaling factors ranging from 1 to ∼0.6 and average successful exchange rates of ∼25%. The hot zone was defined in order to support the sampling of the opening of the TTR interaction. Specifically, the base pairs^78^ *t*HW A6:U36 and *c*WW G8:C35 (i.e., the four nucleotides constituting the receptor part of the TTR; original PDB numbering used) except the phosphates were placed in the hot zone. At the same time, a stabilizing structure-specific HBfix (sHBfix)^79^ of 2 kcal/mol between the hydrogens and acceptors was used to stabilize the H-bonds constituting these base pairs. The sHBfix was not scaled along with the replicas. This setup ensured the REST2 scaling effectively enhances sampling of the tertiary interactions that the receptor forms with the GAAA TL while the sHBfix prevents disruptions of the base pairing H-bonds of the base pairs within the hot-zone; see Supporting Information Text and Figure S2 for further explanation and visualization of the hot zone. All REST2 simulations were carried out with the pmemd.cuda.MPI module in AMBER 22 for 2 μs. The simulation parameters matched those of the standard simulations described above, with the exception that the production phase of the REST2 simulations was conducted in the constant volume (NVT; canonical) ensemble. In one of the OPC hTTR simulations, we also additionally applied mild gHBfix (general HBfix)^22^ correction, specifically counteracting the OPC-induced destabilization of the TTR interactions. Its derivation is explained in Results and Discussion, with further technical details provided in Supporting Information. The REST2 simulations using the DES-Amber FF and TIP4PD water model were performed in GROMACS2018 in combination with PLUMED2.5 ^80^ using the Hamiltonian replica exchange implementation.^81^ The REST2 GROMACS protocol matched the hot-zone definition and the application of the stabilizing sHBfix potential used in the AMBER calculations. However, due to technical limitations prohibiting an odd number of replicas, eight replicas had to be used. In this case, the scaling factor (λ) values ranged from ∼1.07 to ∼0.60, with the second replica serving as the reference (scaling factor 1; unbiased), with an exchange rate of ∼30% throughout the ladder. An additional eighth replica was placed at the bottom rather than the top of the ladder to avoid biasing the TTR toward instability more in GROMACS simulations than in those performed in AMBER.

### Simulations of Small Model Systems with Mixed Water Boxes

To directly estimate the affinity of the different water models to the RNA solute atoms, we constructed systems with mixed water boxes containing equimolar amounts of waters of different models along with a single nucleoside. To allow a direct comparison, the OL3 FF was utilized in each case to describe the nucleoside. To reduce sampling uncertainty, the N-glycosidic angle of all nucleosides was restricted to the *anti* conformation by flat-well dihedral restraints with force constant of 300 kcal/mol/rad^2^ and a fully unbiased range of 150° to 260°. The systems were constructed in xLeap by adding an even number of OPC, TIP4PD, OPC3, or TIP3P water molecules. The number of added water molecules ranged between 1800 and 2050, depending on the system. This was followed by using parmed to turn exactly half of the water molecules into the SPC/E parametrization, by altering all the differing parameters and deleting superfluous atoms if present. For the OPC–SPC/E combination, we performed calculations on all four standard RNA nucleosides as well as on dimethyl phosphate. The dimethyl phosphate structure was generated by excising the phosphate group from a standard nucleotide and capping it with methyl groups, with manually adjusted atomic charges of 0.055800 and 0.049600 for the carbon and hydrogen atoms, respectively. Although this approach is not formally rigorous, we adopted it to ensure that the heavy-atom charges in the phosphate moiety matched those in the standard nucleotides. No ions were included in the nucleoside simulations, whereas a single potassium counterion described by Li–Merz (LM) parameters^71^ for OPC was used in the dimethyl phosphate simulations. For the combinations involving the TIP4PD, OPC3, or TIP3P water models, only the cytidine nucleosides were simulated. Three standard MD simulations were performed for each system, and we calculated the populations of H-bonds formed by the two mixed water models and the H-bond donors and acceptors of the nucleosides or the phosphate. The mixing of the two water models was very carefully monitored and noted to occur already during the equilibration phase of each calculation.

### Analyses

Analyses and visualization of the MD trajectories were carried out using cpptraj and VMD software packages.^82,83^ Graphs were generated with gnuplot, while molecular renderings were created using povray. Conformational transitions in the simulated systems were primarily identified through visual inspection of the simulation trajectories, since we monitored large conformational changes (disruption of tertiary interactions). See also Supporting Information for definitions of observables that can be used to detect the disruptions in each of the systems. In most cases, the structural changes were sufficiently extensive and visually prominent that standard RMSD analysis relatively to the experimental structure was fully adequate to capture the differences observed for the different water models. We also calculated the radius of gyration of the RNA heavy atoms, which showed trends highly consistent with the RMSD analysis (Supporting Information, Figure S3). Additional analysis method was evaluation of the presence of selected tertiary H-bonds based on the interatomic distance and angle of the donor and acceptor groups. Distances between heavy atoms under 4.0 Å and donor-hydrogen-acceptor angles above 120° were considered as cutoff for the H-bonds. Analyses of the REST2 trajectories were performed on the reference (unscaled) replica, as well as on demuxed continuous trajectories. All the replicas and their exchanges along the ladder were monitored. The protein–RNA interface size in the L1 stalk system was calculated by subtracting the solvent-accessible surface area^84^ of the complex from the sum of the individual RNA and protein surface areas. For systems determined by NMR spectroscopy, agreement with the experimental NOE distances (*r*) was assessed by calculating the *r* ^–6^-weighted average of the simulated NOE distances.

## Results and Discussion

### Basic Overview of the Simulations

Below we present results of nearly half a millisecond of MD simulations, comparing the performance of different water models for three RNA systems stabilized by long-range tertiary interactions: two variants of the L1 stalk rRNA, one of them also with the bound L1 protein, the miniTTR-6 construct with a specific fold and one TTR interaction, and the hTTR duplex homodimer with two symmetrical TTR interactions (see Methods and Table 1). The primary metric used for evaluation was the preservation of native RNA folds observed in the experimental structures.

**Table 1:** List of MD Simulations^a^

force field RNA/protein	water model	ion parameters	No. of simulations × length (μs)	time of RNA disruption^b^ (μs)
L1 stalk protein/RNA complex (T.t.)				
OL3/ff14SB	SPC/E	JC	3 × 3	-, -, -
OL3/ff14SB	OPC	LM	3 × 3	0.9, -, -
OL3/ff19SB	OPC	LM	3 × 3	2.9, 2.1, -
OL3_CP_/ff19SB	OPC	LM	3 × 3	1.1, 2.8, -
OL3/ff14SB	TIP3P	JC	3 × 3	-, -, -
OL3/ff14SB	TIP4PEW	JC	3 × 3	-, -, -
OL3/ff14SB	OPC3	LM	3 × 3	-, -, -
DES-Amber	TIP4PD	CHARMM22	3 × 2	0.1, 1.4, 0.8
L1 stalk rRNA (T.t.)				
OL3	SPC/E	JC	3 × 3	-, -, -
OL3	SPC/E	LM	3 × 3	-, -, -
OL3	OPC	LM	3 × 3	0.4∼2.9, -, -
OL3	OPC	JC	3 × 3	0.8∼1.0, 0.7∼2.4, -
OL3_CP_	OPC	LM	3 × 3	1.7∼1.9, 2.4, -
OL3	TIP3P	JC	3 × 3	-, -, -
OL3	TIP4PEW	JC	3 × 3	-, -, -
OL3	OPC3	LM	3 × 3	-, -, -
L1 stalk rRNA (H.m.)				
OL3	SPC/E	JC	3 × 3	-, -, -
OL3	OPC	LM	3 × 3	0.5, 0.1, 0.4
OL3	TIP3P	JC	3 × 3	-, -, -
OL3	TIP4PEW	JC	3 × 3	1.4, -, -
OL3	OPC3	LM	3 × 3	1.5∼1.9, -, -
DES-Amber	TIP4PD	CHARMM22	3 × 1	0.1, 0.2, 0.1
miniTTR-6				
OL3	SPC/E	JC	4 × 10	-, -, -, -
OL3_CP_	OPC	LM	3 × 10	1.2, 1.6, 1.5
OL3_CP_ ^c^	OPC	LM	3 × 10	0.3, 0.1, 0.1
OL3_CP_ ^d^	OPC	LM	4 × 10	3.8, 0.4, 0.7, -
OL3^e^	SPC/E	JC	1 × 5, 3 × 10	0.0∼4.2, 0.0∼2.0, 0.0∼0.4, 0.0∼0.2
DES-Amber	TIP4PD	CHARMM22	3 × ∼1.7	0.8, 0.1, 0.7
DES-Amber^c^	TIP4PD	CHARMM22	3 × 3	0.2, 2.0, 0.7
DES-Amber^d^	TIP4PD	CHARMM22	3 × 3	0.8, 0.4, 0.6
DES-Amber^e^	TIP4PD	CHARMM22	1 × 4.4	0.0∼4.4
hTTR				
OL3	SPC/E	JC	3 × 10	-, -, -
OL3	OPC3	LM	3 × 10	-, -, -
OL3_CP_	OPC	LM	4 × 10	8.0, 8.2, 5.3, -
DES-Amber	TIP4PD	CHARMM22	4 × 10	0.1, 1.3, 0.1, 0.9
OL3 (REST2)	SPC/E	JC	7 × 2	none
OL3 (REST2)	OPC3	LM	7 × 2	none
OL3_CP_ (REST2)	OPC	LM	7 × 2	many^f^
DES-Amber (REST2)	TIP4PD	CHARMM22	8 × 2	many^f^
OL3 (REST2)	TIP4PD	CHARMM22	7 × 2	many^f^
OL3_CP_ ^g^ (REST2)	OPC	LM	7 × 2	none
Small model systems with mixed water boxes^h^				
OL3	SPC/E + OPC	-	3 × 10 (cytidine)	N/A
			3 × 10 (uridine)	
			3 × 10 (guanosine)	
			3 × 10 (adenosine)	
			3 × 10 (phosphate)	
OL3	SPC/E + OPC3	-	3 × 10 (cytidine)	N/A
OL3	SPC/E + TIP4PD	-	3 × 10 (cytidine)	N/A
OL3	SPC/E + TIP3P	-	3 × 10 (cytidine)	N/A

a See Supporting Information, Table S1 for simulations of additional RNA systems.

b Indicates the simulation time at which a characteristic disruption of the native RNA fold was observed; see Results and Discussion below for the description of what constitutes the disruption in each of the systems. See also Supporting Information for definitions of observables that can be used to detect the disruptions. Multiple values refer to the different replicate trajectories. Values shown as a range “∼” indicate the time interval during which a reversible disruption occurred (i.e., the time period during which the tertiary contact was broken in a given simulation), whereas single values mark a disruption irreversible on the simulation time scale. The “-” mark indicates no disruptions were observed for a given replicate trajectory. Note that due to the relatively small number of simulations the reported disruption times should not be interpreted quantitatively, but rather as a qualitative indication of whether a given water model exhibits RNA-fold disruptions.

c All nine crystallographically determined bound Mg^2+^ ions were included in the simulation (see the Methods); this simulation condition is referred to as “+Mg^2+^” in the figures, and is used together with the standard 0.15 M excess KCl.

d Bulk 0.04 M Mg^2+^ ion concentration was used, in addition to all the crystalographically determined bound Mg^2+^ ions, together with the standard 0.15 M KCl (see the Methods); this simulation condition is referred to as “++Mg^2+^” in the figures.

e A snapshot from one of the OL3/OPC simulations where the structure became disrupted was used as the start (Supporting Information, Figure S4).

f Numerous disruptions were observed in the basic (reference) replica and also across the demuxed trajectories.

g Corrections derived from simulations of the small model systems were implemented as stabilizing gHBfix (general HBfix) potentials,^22,28^ suppressing the OPC-induced instability (see below). For detailed explanation and parameters of the used gHBfix modification, see Supporting Information.

h Simulations of isolated nucleosides or dimethyl phosphate in equimolar mixtures of the two water models. See Methods and details below.

For all three tested systems, the four-point OPC and TIP4PD water models were universally less successful in maintaining the native folds, with both reversible and irreversible disruptions observed that were not seen with the other tested water models. We note that use of the adjusted phosphate van der Waals (vdW) parameters by Steinbrecher et al.^20^ along with the OL3 FF (what we term as the OL3_CP_ variant) did not prevent the loss of the native RNA folds in the OPC simulations. The OL3 and OL3_CP_ RNA FF variants provided identical performance within the limits of our sampling (Table 1) and they will be henceforth not described separately. For some systems, we subsequently tested only the OL3_CP_ variant in combination with OPC, as it has been suggested to perform better with this water model in small RNA systems^2,85^ and is used as such in most published studies. Likewise, cross-testing of the JC and LM monovalent ion parameter (see Methods) sets showed that they did not affect either the loss of native RNA folds in the OPC simulations or the generally good performance observed in the SPC/E simulations (Table 1). In conclusion, the OPC water model appears to be the sole factor responsible for the structural instabilities in the OL3/OPC simulations of the tested systems.

Qualitatively identical instabilities to those seen in the OL3/OPC simulations were also observed with DES-Amber/TIP4PD FF combination. One notable difference came from the apparent tendency of the DES-Amber FF to favor extended A-form-like structures. Namely, once the TIP4PD water model initially “loosened” the native fold, additional structural rearrangements immediately followed, driving the structure even further away from the native fold and preventing its restoration. In contrast to the OL3/OPC simulations, where disruptions were sometimes reversible and the native fold could at least temporarily recover, no such restorations were ever observed with DES-Amber/TIP4PD on our simulation time scale. We suggest that this outcome is primarily attributable to genuine differences between DES-Amber and OL3 RNA FFs; in a recent study we noted that DES-Amber FF (as well as its predecessor DESRES^86^) are somewhat biased in favor of the A-form-like RNA structures.^18^ However, when focusing solely on the structural influence of the water models (the topic of this paper), TIP4PD and OPC exert essentially identical destabilizing effects on the native RNA folds.

### L1 rRNA Undergoes Structural Disruption in OPC Simulations

Isolated L1 rRNA from both *T.t.* and *H.m.* as well as the L1 stalk protein-RNA complex from *T.t.* exhibited reduced structural stability when simulated with the OPC water model (Figure 1 and Table 1). Among the three structures, the isolated L1 stalk rRNA from *H.m.* underwent the most rapid structural degradation, with permanent loss of its structure in entirely all OPC simulations as well as even in one simulation with the TIP4PEW model (an older four-point water model from 2004).^36^ The L1 stalk rRNA from *T.t.*, whether in isolation or complexed with the L1 protein, was more resistant to structural changes, showing disruptions in only some OPC simulations. In the simulations of the isolated L1 stalk rRNA, the disruptions were additionally reversible on the simulation time scale with one exception (Table 1). In contrast, the DES-Amber/TIP4PD simulations exhibited rapid and irreversible disruption of the L1 stalk in both the isolated rRNA and the protein-RNA complex. Moreover, the structural rearrangements were substantially more extensive (Figure 1C,D), in some cases necessitating early termination of the simulations to avoid solute image clashes. It should be noted that in an earlier study,^38^ full stabilization of the L1 stalk complex in DES-Amber/TIP4PD simulation was achieved through the inclusion of high concentration of bulk Mg^2+^ ions.

**Figure 1:**
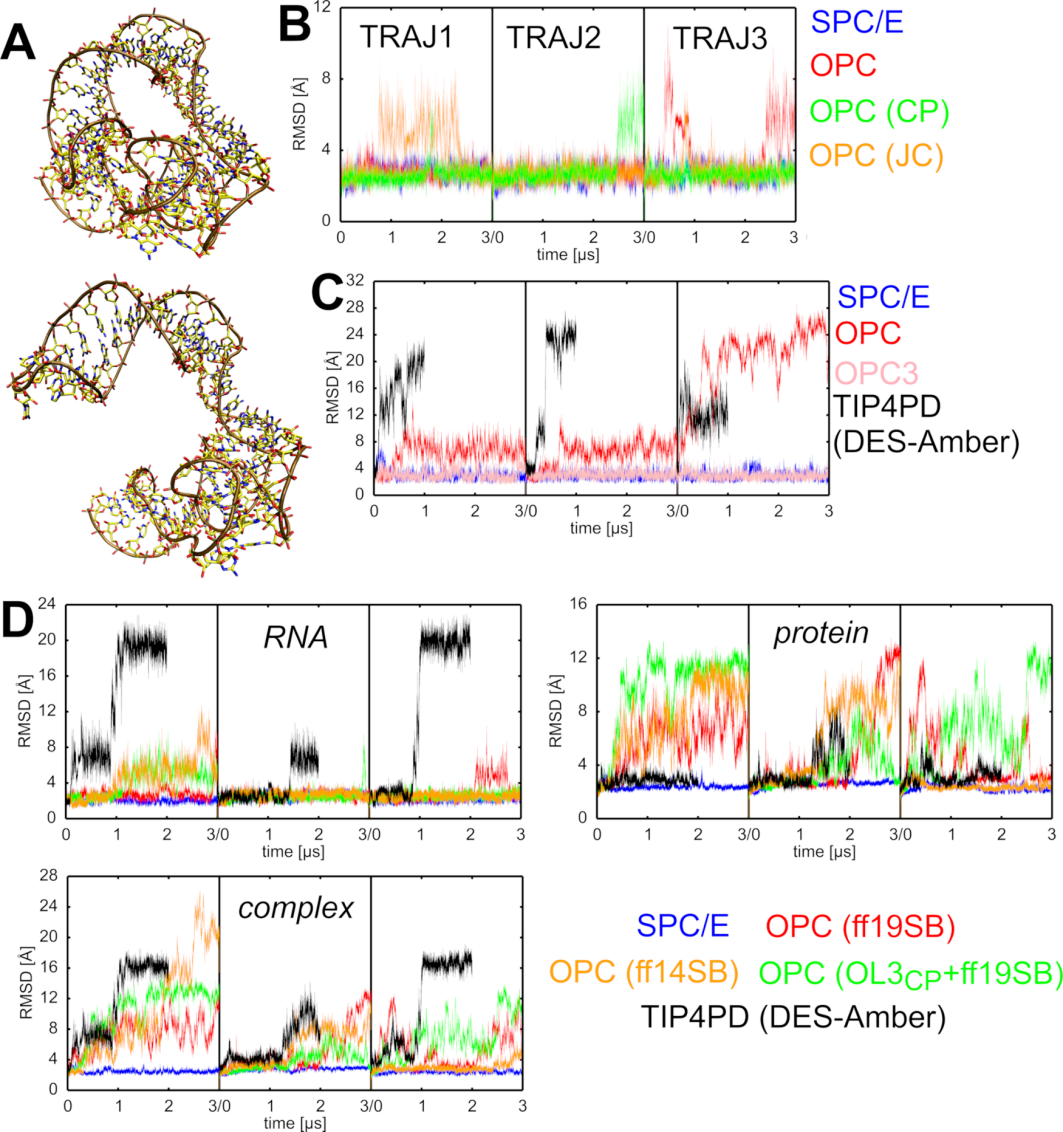
Loss of the native RNA fold in simulations of L1 stalk rRNA. (A) Comparison of the experimental structure (top) and an example of a disrupted structure typically seen in OPC and TIP4PD simulations (bottom). More extended A-form-like structures could be observed with the DES-Amber/TIP4PD FF combination (Supporting Information, Figure S5). (B) Time evolution of the RMSD of the isolated L1 stalk rRNA from *T.t.* in individual simulations using selected water models. Data sets are color-coded according to the legend on the right (see also Table 1). For space saving reasons, all three independent parallel simulations (replicates) per water model are shown in a single plot, with individual trajectories separated by black vertical lines. The time evolution graphs will be presented in this manner henceforth. (C) Same as B, but for the isolated L1 stalk rRNA from *H.m.* (D) Time evolution of the RMSD of the L1 stalk protein–RNA complex from *T.t.* Separate RMSD plots are shown for the RNA, the protein, and the entire complex.

Overall, the three-point water models showed higher stability for the L1 stalk system, with merely one short reversible disruption observed for the *H.m.* system (Table 1).

### Collapse of the Protein–RNA Interface of the L1 Stalk in OPC Water Simulations

In addition to the rRNA disruptions described above, we consistently observed a gradual major loss of the protein–RNA interface in the OPC water model simulations (Figure 2). In some of the trajectories, the L1 protein almost entirely disengaged from the rRNA by the simulation end. Notably, these changes appeared to be independent from the RNA disruptions (see above) and were even faster, suggesting a distinct and separate issue attributable to the water model (Figure 2B). The primary loss of the RNA structure occurs rather far from protein binding area. To assess whether the protein–RNA interface instabilities could be related to the utilized protein FF, we tested both the ff14SB and ff19SB protein FFs. We observed the loss of the interface occurring with both FFs in the same manner. Note that the ff19SB is primarily recommended to be used with the OPC water model by the authors.^30^ The protein–RNA interface is unstable also with the DES-Amber/TIP4PD FF combination (Figure 2B). In contrast, simulations using three-point water models such as SPC/E and OPC3 did not reveal any significant destabilization of the protein–RNA interface or the RNA fold (see above) in the L1 stalk system.

**Figure 2:**
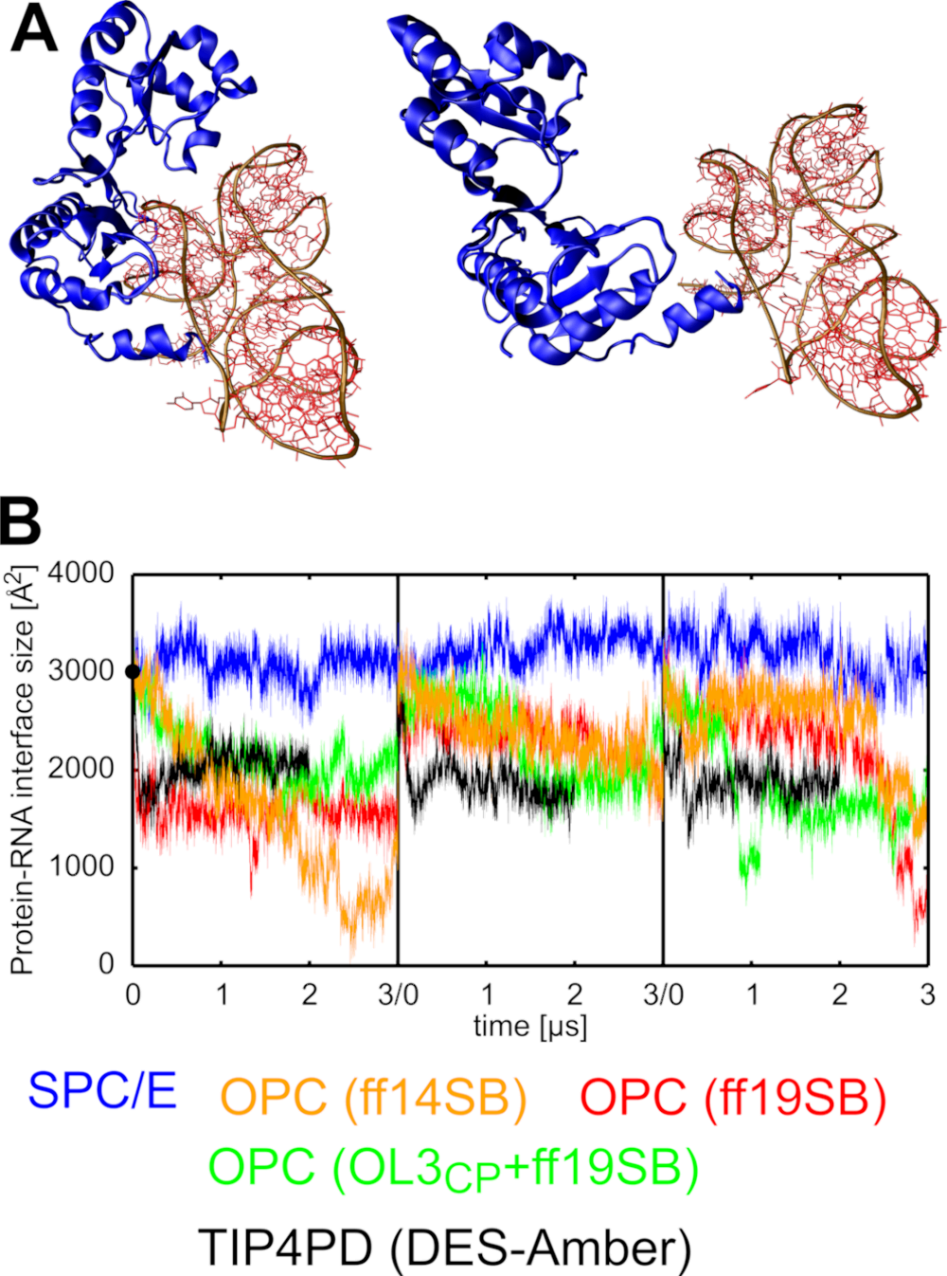
Destabilization of the protein-RNA interface in L1 stalk simulations. (A) Comparison of the experimental structure (left) and a representative structure from OPC simulations (right), showing the L1 protein nearly fully disengaged from the rRNA. Protein domain is shown as blue ribbons, RNA atoms in red, and the RNA backbone in brown. (B) Time evolution of the protein–RNA interface size in individual simulations using selected water models. The black circle on the *Y*-axis indicates the experimental value. Data sets are color-coded according to the legend below the graphs.

### OPC Water Model Induces Large-Scale Unfolding of the miniTTR-6 System

A certain limitation of the L1 stalk system (see above) is that the OPC-induced structural collapse cannot be easily attributed to a well-defined set of problematic solute–solute interactions. Instead, the system appears to undergo diverse and multistep structural rearrangements prior to the structural disruption that furthermore does not occur in every simulation. Nevertheless, among the affected interactions, we noticed those involving RNA 2′-OH groups as particularly sensitive and usually the first to locally disappear prior to the more global changes. To examine this in a more focused manner, we next selected the miniTTR-6 system, which among other noncanonical structural features (i.e., a kink-turn and IRES element) contains a long-range TTR – a motif known to be stabilized by an extensive network of 2′-OH-mediated interactions (Figure 3).

**Figure 3:**
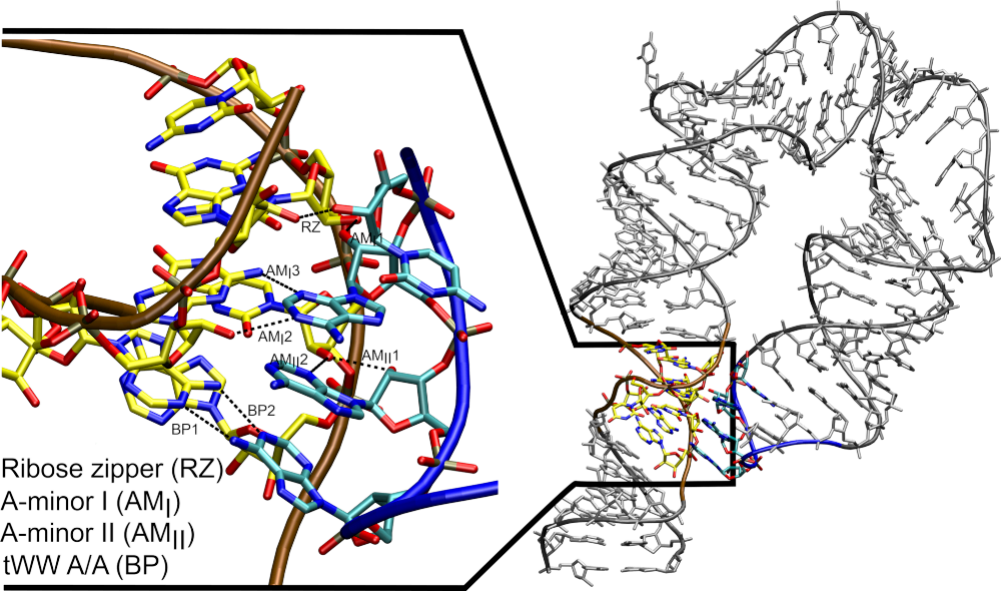
Structural overview of the GAAA tetraloop–tetraloop receptor (TTR) motif within the miniTTR-6. The TTR motif can be divided into four noncanonical interaction submotifs, which are depicted in the figure. The tetraloop receptor and GAAA TL parts of the motif have their carbon atoms colored in yellow and cyan and their backbone trace in brown and blue, respectively. Individual H-bonds are indicated by black dashed lines and labeled using motif-specific abbreviations. Multiple H-bond interactions within the same submotif are given a numerical suffix to differentiate them. This labeling scheme is applied consistently throughout the present study. Topologically identical TTR motifs are found in the miniTTR-6 and hTTR systems.

Simulations of miniTTR-6 using the OPC water model showed rapid disruption of the TTR motif (Figure 4 and Table 1). This was sometimes followed by a straightening of the entire structure. No such disruptions were observed when using the SPC/E water model. We also tested the possible stabilizing influence of the divalent ions by performing the OPC simulations under three different ionic conditions: (1) standard 0.15 M excess KCl concentration, (2) inclusion of the nine crystallographically resolved Mg^2+^ ions (see Methods), and (3) inclusion of both experimental Mg^2+^ ions together with a 0.04 M concentration of bulk Mg^2+^ ions (see the Methods and Table 1). The 0.04 M bulk Mg^2+^ concentration was selected for testing because it has previously been reported to stabilize the L1 stalk structure in DES-Amber/TIP4PD simulations,^38^ and we wished to determine whether it might produce a similar effect for miniTTR-6. Both magnesium conditions were supplemented by 0.15 M KCl. However, in all cases, the same TTR disruption was observed (Figure 4 and Table 1), reinforcing the conclusion that the destabilizing effect is primarily attributable to the OPC water model. Notably, when a structure previously disrupted by OPC was resimulated in a larger SPC/E water box with a standard KCl concentration of 0.15 M, the native fold was fully restored within ∼4.2 μs (Figure 5). We subsequently performed three additional such simulations, which also resulted in full restoration in two cases and a partial restoration in the third (Supporting Information, Figure S6). These amazing recoveries highlight that the OL3 FF, when paired with SPC/E water, is eventually capable of reversing the OPC-induced damage, at least in some cases. Importantly, the result pinpoints the water model as the prime factor in the observed destabilization and indicates that the basic OL3 FF, albeit being far from flawless, likely provides rather reasonable description of many folded RNAs.^18^

**Figure 4:**
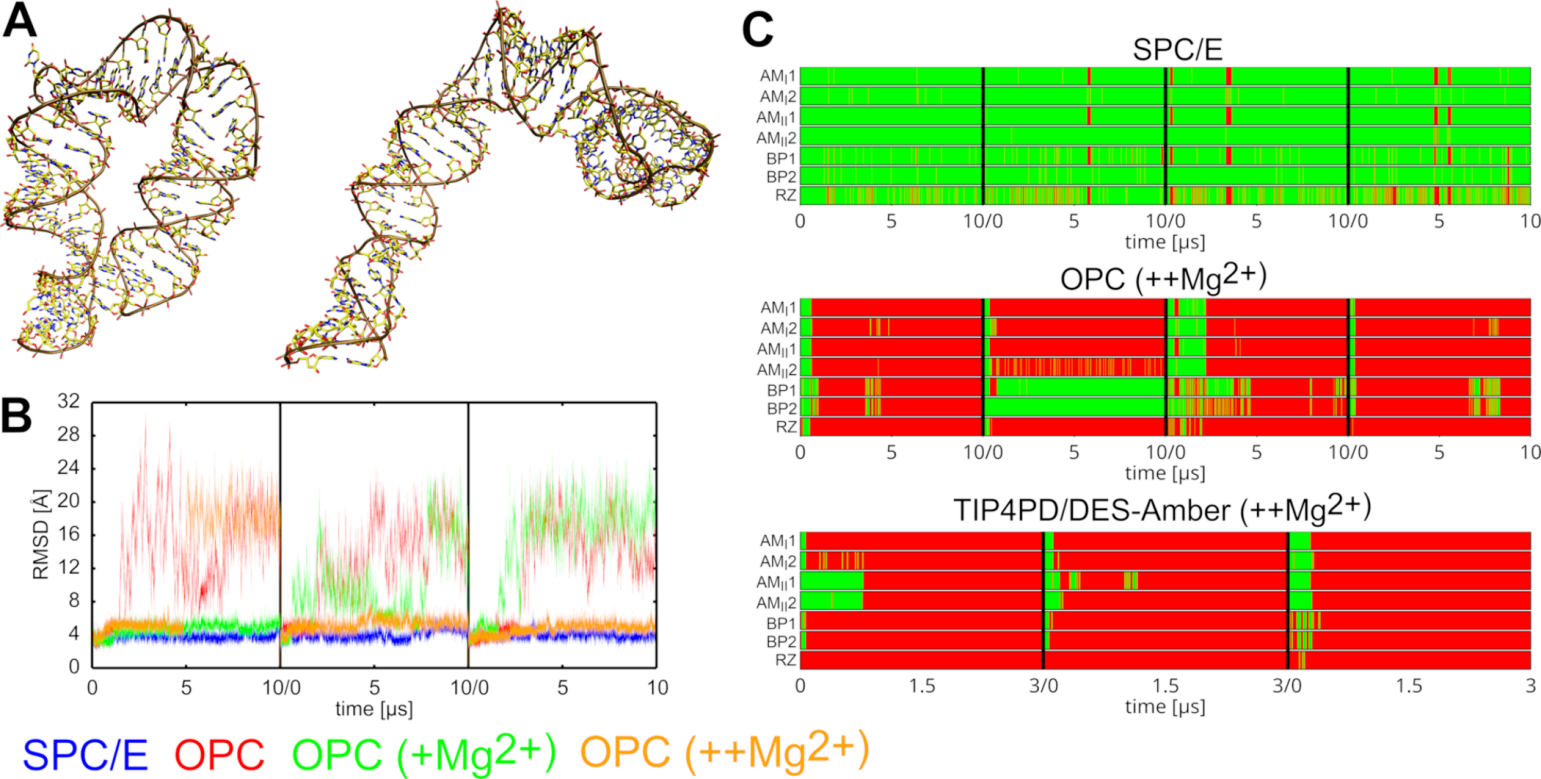
Loss of the native RNA fold in simulations of miniTTR-6. (A) Comparison of the experimental structure (left) and a selected representative structure from OPC simulations (right). (B) Time evolution of the RNA RMSD in selected simulations using different water models and ionic conditions (see Methods). Data sets are color-coded according to the legend at the bottom (see the footnotes of Table 1 for explanation of the abbreviations used to define the Mg^2+^ conditions). See also above for a discussion of the effects of using the OL3 versus OL3_CP_ FF variants. (C) Time evolution of the key TTR H-bonds in selected simulations using different water models. Green and red indicate presence and absence of the H-bond, respectively. See Figure 3 for definition of the individual H-bonds. It is notable that the inclusion of Mg^2+^ ions does not stabilize the structure in the simulations. According to the experiments, the miniTTR6 structure folding is greatly aided already by very low concentrations of Mg^2+^, but in principle it folds also in excess of monovalents.^52^

**Figure 5:**
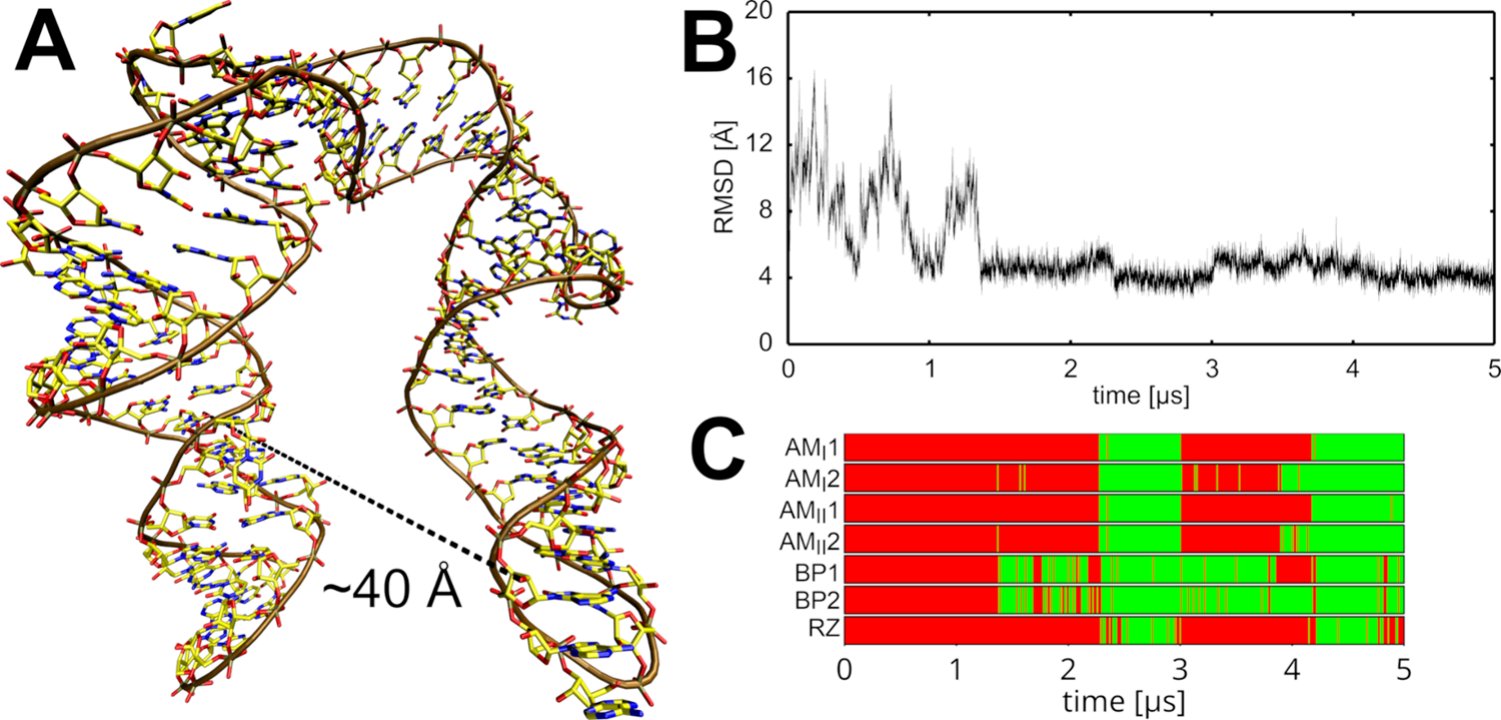
Restoration of the native fold of miniTTR-6 in SPC/E simulation. (A) A structure previously disrupted in OPC simulations, with the TTR binding partners positioned far apart. (B) Time evolution of the RNA RMSD in the restoration simulation using the SPC/E water model. (C) Time evolution of the signature TTR H-bonds. Green and red indicate presence and absence of the H-bond, respectively. See Figure 3 for definition of the individual H-bonds. See also Supporting Information, Figure S6 for additional replicates of these simulations.

### DES-Amber/TIP4PD Setup Exhibits Similar Instabilities in miniTTR-6 Simulations as the OL3/OPC Combination with an Additional Straightening of the Structure

In our present simulations of the miniTTR-6 system, all the DES-Amber/TIP4PD simulations consistently led to the disruption of the TTR motif (Figure 4C and Table 1), followed by extensive global rearrangement (Supporting Information, Figure S7). Similar to the OL3/OPC combination, this occurred regardless of the inclusion of the Mg^2+^. In contrast to OL3, where we showed the OPC-disrupted structures could be restored by transferring them into SPC/E water, the same approach could not be tested with DES-Amber. This is because DES-Amber incorporates solute–solvent NBfix modifications specifically tailored for TIP4PD,^38^ rendering it incompatible with other water models. Nevertheless, we have enlarged the water box to eliminate any image clashes and potentially allow the system to spontaneously relax back to the native conformation. However, no such restoration was observed. Instead, the system exhibited a clear tendency to deviate further from the native fold, which indicates potential bias in the RNA FF toward the A-form (Supporting Information, Figure S8).

### Simulations of the GAAA Tetraloop–Tetraloop Receptor (hTTR) Homodimer Complex Consistently Reveal a Loss of One TTR

As the final primary test system, we selected a construct composed of two symmetrical A-RNA helices forming a homodimer via two identical TTR motifs. Unlike the miniTTR-6 system, which contains additional recurrent motifs that could contribute to the structural changes in some fashion, the hTTR construct effectively isolates the canonical TTR motifs (Figure 3) in a minimal but experimentally well-defined context.^51^ Indeed, in our simulations, the hTTR construct exhibited overall greater structural stability than miniTTR-6, supporting our hypothesis that additional structural strain in miniTTR-6 might be influencing the disruption events. Nevertheless, simulations using the OPC water model still displayed signs of instability. Namely, in two out of four OL3/OPC simulations, one of the two TTRs was irreversibly disrupted (Table 1 and Figure 6), while the second TTR remained stable over the course of the simulation. In a third simulation, an irreversible partial disruption of both TTRs was observed. Likewise, the DES-Amber/TIP4PD simulations revealed the loss of one TTR in all four trajectories on even a shorter time scale than the OL3/OPC (Table 1 and Figure 6). In contrast, no such prolonged TTR disruptions were observed in simulations using the SPC/E or OPC3 water models, conclusively showing the four-point OPC and TIP4PD water models as the destabilizing factor.

**Figure 6:**
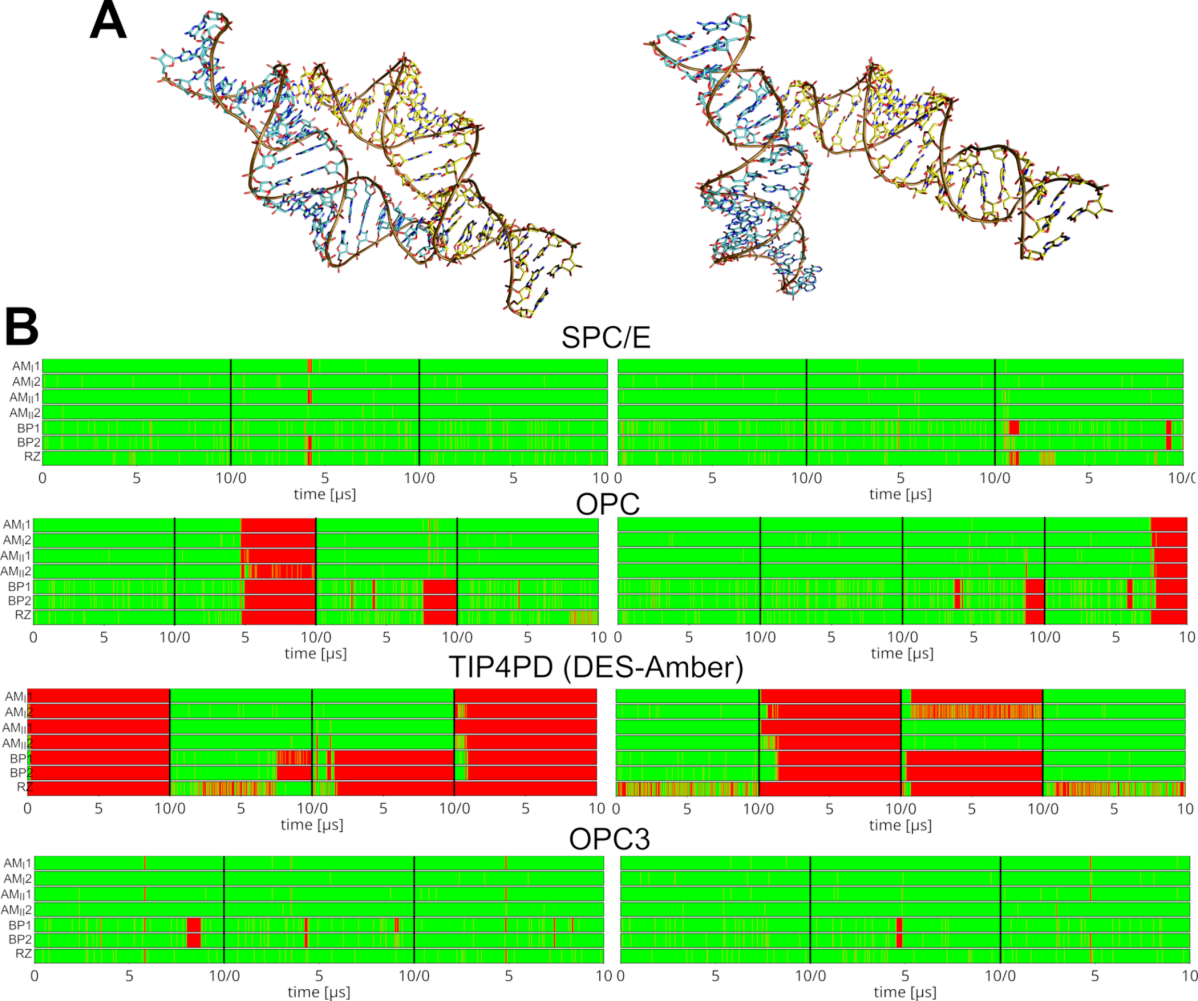
Loss of the native RNA fold in standard MD simulations of the hTTR. (A) Comparison of the experimental structure (left) and a typical structure observed in simulations using the OPC or TIP4PD water models where only one of the two TTRs remains intact (right). The two helices forming the hTTR have their carbons colored in yellow and cyan, respectively. (B) Time evolution of the signature TTR H-bonds (see Figure 3) in simulations using different water models. Green and red indicate the presence and absence of the H-bond, respectively. The left and right graphs show the first and second TTR site in the hTTR system, respectively.

### REST2 Enhanced Sampling Simulations Confirm OPC- and TIP4PD-Induced Destabilization of TTR

In standard MD simulations of the hTTR system using the OPC or TIP4PD water models, only one out of the two TTR motifs was fully disrupted on the time scale of our simulations (see above and Figure 6). In addition, for the OL3/OPC combination, the disruption events occurred relatively late in the simulations. Nevertheless, the lesser stability of the OPC and TIP4PD simulations was evident, even though a fully converged picture was not obtained.

To further examine this, we next performed REST2 enhanced sampling simulations of the hTTR using the SPC/E, OPC, OPC3, and TIP4PD water models (see also Methods and Table 1). We observed that the TTR motifs were best maintained in simulations using the SPC/E and OPC3 water models (Figure 7 and Table 2). In OPC REST2 simulations we observed some TTR instability, including a simultaneous loss of both TTR motifs in one continuous trajectory (Supporting Information, Figure S9). Note that in standard MD simulations, we could always observe only the loss of a single TTR motif. All the disruptions observed in the OPC REST2 simulations were fully reversible on the simulation time scale, as the system was intermittently switching between the disrupted and bound states at the two ends of the homodimer. Even when both TTR motifs were transiently lost and the two helices briefly drifted apart randomly in the box in one continuous trajectory, the native interface was eventually reformed over time (Supporting Information, Figure S9). Note that, at first sight, the OPC simulations appear to perform better in REST2 than in the standard MD, where only irreversible TTR disruptions were observed. However, we suggest that this apparent difference reflects the fact that the 10 μs time scale of the standard MD simulations was not sufficient to capture the TTR reintegration events. We anticipated such a sampling limitation for this system, which motivated the use of REST2 simulations to obtain a more converged picture of the hTTR dynamics. We suggest the REST2 calculations conclusively show the OPC-induced destabilization of the hTTR structure while also demonstrating the good performance of the OL3 RNA FF for the TTR interaction. This result contrasts sharply with the DES-Amber/TIP4PD REST2 simulations, which showed only irreversible losses of the TTR, with simultaneous loss of both TTRs having occurred in over half of the continuous trajectories by the simulation end. To disentangle the effect of the solute FF from that of the water model, we performed additional REST2 simulations with the nonstandard OL3/TIP4PD combination (Table 1). In this setup, performance for the hTTR improved markedly, still remaining somewhat inferior to OL3/OPC, but comparable (Figure 7 and Table 2). Lastly, note that loss of one or both TTR motifs, as observed with the OPC and TIP4PD water models, is also associated with an increased number of experimental NOE distance violations (Supporting Information, Table S2).

**Figure 7:**
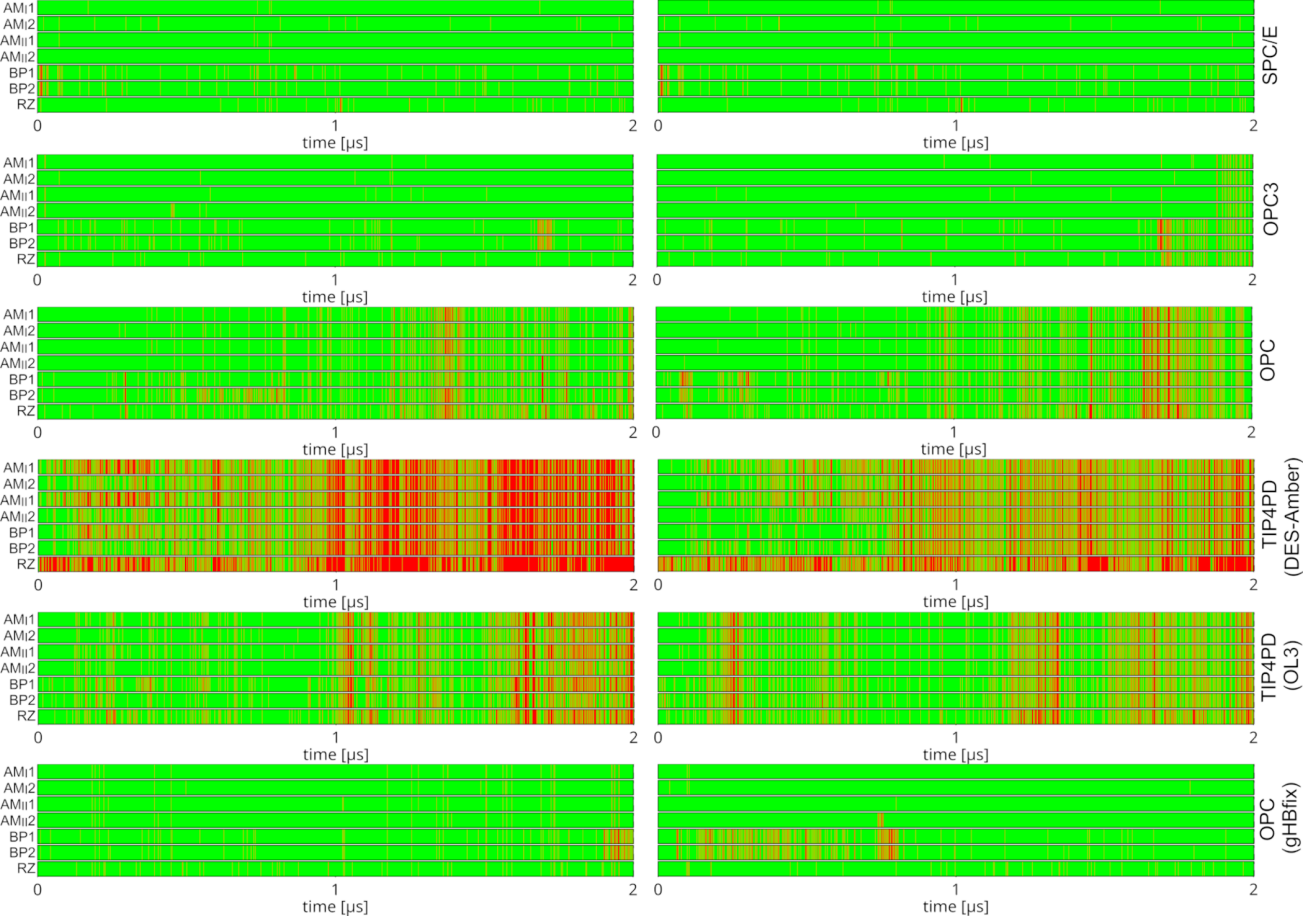
REST2 enhanced sampling simulations of the hTTR. Time evolution of the signature TTR H-bonds in the unscaled replica of REST2 simulations using different water models. Green and red indicate the presence and absence of each H-bond, respectively. See Figure 3 for definitions of individual interactions. Left and right graphs show the first and second TTR site in the hTTR system, respectively.

**Table 2:** Populations (in %) of Conformational States Sampled in REST2 Simulations of the hTTR^a^

water model/conformation	both TTRs stable	single TTR stable	no TTR stable
SPC/E	100	0	0
OPC3	96	4	0
OPC	62	38	0
DES-Amber/TIP4PD	0	75	25
OL3/TIP4PD	51	34	15
OPC + gHBfix^b^	98	2	0

a The hTTR system contains two TTR motifs, either of which could become disrupted in the REST2 simulations. Reported values correspond to the last 500 ns of the unscaled (basic) replica. The presence of a TTR interaction was evaluated by measuring the distance between the centers of geometry defined by the tetraloop and tetraloop receptor H-bonding heavy atoms, respectively. Distances between the centers greater than 12 Å were classified as disrupted motifs (i.e., the TTR not present). By measuring this distance for both TTR motifs, we classified the ensemble states into three categories: both TTRs present, only one TTR present (either of them), or no TTR present.

b The gHBfix potentials specifically tuned to suppress OPC-induced destabilization were applied (see below and Supporting Information).

### OPC and TIP4PD Waters Preferentially Interact with RNA H-Bond Donor/Acceptor Groups Relative to SPC/E

Detailed inspection of all trajectories of the tested systems suggested that the OPC and TIP4PD waters destabilize the RNA structures by excessively competing against native RNA–RNA H-bonding. In other words, H-bond donors and acceptors in RNA seem to form more frequent interactions with the tested four-point water models compared to the three-point models, which might explain the observed disruptions of the native intramolecular interactions essential for the RNA structural integrity. While the trend is qualitatively clear, quantifying the extent of this “water insertion” interference for the large RNA systems is challenging due to sampling limitations, presence of numerous such interactions and complexity of the conformational changes. For example, it is not feasible to determine the extent to which the water model interacts with individual atomic groups and calculate the free energy, as the systems are too large to obtain sufficiently converged trajectories for every single H-bond donor and acceptor. In other words, attempting any detailed free-energy decomposition of the individual interactions would not be feasible.

Therefore, in an effort to provide more quantitative insights that can however still be generalized to larger RNAs, we constructed simple nucleoside model systems solvated in equimolar mixtures of two different water models (see Methods). In case of the OPC–SPC/E mixture, the studied systems included all four standard RNA nucleosides. We also constructed a fifth system containing a dimethylphosphate to estimate the water affinity difference for the phosphate group. For the other tested water model mixtures (OPC3–SPC/E, TIP4PD–SPC/E, and TIP3P–SPC/E), only systems containing the cytidine nucleoside were explored. All the trajectories were subsequently analyzed to calculate the relative populations with which the different water models interact with the individual H-bond donors and acceptors. The rationale is that the relative occupancies of each water model at specific RNA donor or acceptor sites should reflect their relative affinities for those sites. Unlike in the large RNA systems, for the small model systems the simulations converge rapidly and the occupancies can be determined with full confidence. A deviation from the 1:1 binding ratio then indicates preferential binding by one water model to the site which can be expressed as free energy difference using the Boltzmann two-state model.

We show that the OPC water molecules exhibit higher affinity for entirely all RNA H-bond donors and acceptors than the SPC/E water molecules (Table 3). A very similar trend was observed when comparing TIP4PD with SPC/E (Supporting Information, Table S3). In contrast, comparisons of SPC/E with TIP3P and OPC3 revealed more balanced behavior. Specifically, TIP3P interacted more strongly with the acceptor groups but more weakly with the donor groups than SPC/E, resulting in an overall compensating net effect. For OPC3, the differences in affinity toward both donors and acceptors relative to SPC/E were minimal (Supporting Information, Table S3). While the differences per single H-bond donor or acceptor shown in Table 3 and Supporting Information, Table S3 are quite small, they may become significant for large RNAs where many such interactions form and collectively contribute to the stability of the native RNA folds.

**Table 3:** Difference in Estimated Binding Free Energy (in kcal/mol) between the OPC and SPC/E Water Molecules for Specific H-Bond Donors and Acceptors of Standard RNA Nucleosides and the Phosphate Group^a^

RNA atom	cytidine	uridine	guanosine	adenosine	phosphate^b^
Acceptors					
O5′	0.06	0.06	0.07	0.06	0.13
O2′	0.11	0.10	0.11	0.10	
O3′	0.08	0.08	0.08	0.08	0.13
O2	0.13	0.10			
N3	0.14		0.09	0.07	
O4		0.07			
O6			0.10		
N7			0.10	0.08	
OP1					0.08
OP2					0.08
Donors					
HO5′	0.11	0.11	0.12	0.12	
HO2′	0.12	0.12	0.12	0.12	
HO3′	0.11	0.11	0.11	0.11	
NH41	0.08				
NH42	0.07				
H3		0.10			
H1			0.11		
NH21			0.09		
NH22			0.10		
NH61				0.07	
NH62				0.08	

a Positive number means the RNA donor/acceptor interacts more strongly with the OPC water than with the SPC/E, which is the case for all entries. The values represent combined simulation ensembles of all three simulation replicates. Virtually no variance was observed in the simulations on the time scale of hundreds of nanoseconds, indicating excellent convergence (see Supporting Information, Figure S10). Note that the reported values refer to individual donor or acceptor groups. To estimate the penalization of a specific H-bond in folded RNA systems (see also Supporting Information), the values for the corresponding donor and acceptor must be summed.

b A dimethyl phosphate model was used to estimate the water affinity to the negatively charged phosphate group present in the RNA sugar–phosphate backbone.

### hTTR Structure Can Be Stabilized in REST2 Simulations through the Use of gHBfix

As described above, we calculated the affinity differences between the SPC/E and OPC water models toward the H-bond donors and acceptors in each individual RNA nucleoside and in the phosphate (Table 3). This enabled us to calculate the extent (in kcal/mol) to which the stability of each possible RNA–RNA H-bond is underestimated in OPC simulations. These values can be directly applied as gHBfix^22,28^ coefficients (Supporting Information) and used to stabilize RNA folds when employing the OPC water model. Within the gHBfix framework, individual H-bonds of a given type (e.g., N–H···O) can be globally strengthened or weakened through auxiliary potentials,^79^ the magnitude of which can be flexibly tuned to the specific requirements of each interaction. Thus, in principle, modest gHBfix correction could be used to compensate for the destabilizing influence that the OPC water model exerts on some folded RNAs.

When we applied the stabilizing gHBfix potentials derived from Table 3 to the hTTR system, the resulting REST2 simulations with the OPC water exhibited performance comparable to the simulations using the three-point water models. This reinforces the conclusion that the OPC-induced destabilization of RNA–RNA H-bonds is indeed very small on the level of individual interactions, as indicated in Table 3. However, the cumulative effect can become substantial when many such interactions act in concert, leading to notable structural consequences for some large folded RNAs. These results also suggest that the free-energy values obtained from our mixed water-box simulations (see above and Table 3) are likely reasonably accurate.

### Additional Tested RNA Systems

Based on the referee’s suggestion, we performed additional tests on other RNA systems, the details of which are summarized in the Supporting Information. Briefly, we examined the Sarcin–Ricin Loop (SRL) motif,^87^ the 5S rRNA Loop E,^88^ and the RNA three-way junction.^89^ For the Loop E, we observed no detectable differences between the water models on the time scale of our simulations, suggesting the OPC and TIP4PD water models are not problematic for this duplex containing seven consecutive noncanonical base pairs. For the SRL system, both OPC and TIP4PD exhibited reduced stability of one of the key base-phosphate interactions compared with the SPC/E simulations. The TIP4PD/DES-Amber combination additionally led to irreversible destabilization of the intricate internal loop of the SRL, including the structurally essential GpU platform motif.^87^ This effect has been reported previously^38^ and is likely attributable to the DES-Amber RNA FF rather than the water model itself. Finally, the RNA three-way junction showed roughly comparable performance with the SPC/E and OPC water models (Supporting Information).

## Conclusions

Through an extensive series of MD simulations on three structured RNA systems, we have demonstrated that the OPC and TIP4PD water models can induce structural instability in some folded RNAs. This behavior is documented for the ribosomal L1 stalk, miniTTR-6 construct, and GAAA tetraloop-tetraloop receptor homodimer (hTTR) complex, using a selection of popularly used RNA FFs and ion parameters. In each case, the water model was identified as the primary factor responsible for the observed reduced structural stability of the RNA fold, independent of the RNA FF, ion parameters, or ion concentration. These findings raise some concerns about the increasingly common use of the OPC and TIP4PD water models in RNA simulations, particularly in systems involving complex H-bonding networks and tertiary interactions. The problem extends also to protein–RNA interfaces as loss of the L1 stalk complex interface, the single protein–RNA complex tested in this work, was also observed in simulations using the four-point water models. Analysis of water occupancy around RNA donor and acceptor atoms in simulations of small model systems shows that OPC and TIP4PD water molecules interact with the RNA with greater affinity than SPC/E water, by ∼0.1–0.3 kcal/mol per H-bond. We suggest this eventually allows the water molecules to outcompete some of the native RNA–RNA H-bonds, thus reducing stability of some RNA folds. In small systems such as RNA tetranucleotides or hairpins, this effect can be negligible or even beneficial, as it tends to favor less compact conformations. However, in larger folded RNAs, it may introduce a free-energy imbalance that affects stability of the native folds. Particularly sensitive are interactions where the donors/acceptors are solvent-exposed or locally flexible, such as the 2′-OH groups. This includes the ubiquitous A-minor motif (a consecutive A-minor II and A-minor I interaction), present in the TTR systems tested here and quite common in large RNAs.^53^ At the same time, our results indicate that, in principle, all donor and acceptor groups are affected to a similar extent (Table 3). The heightened sensitivity of 2′-OH groups is therefore likely a kinetic effect arising from their ability to freely rotate and from the frequent native balance between direct and water-mediated H-bonding patterns.

Taken together, our results indicate that the OPC and TIP4PD water models, despite their favorable bulk properties and recent popularity in MD simulations, might not be universally best suited for maintaining the structural integrity of some folded RNAs or even protein-RNA complexes. For at least some of the folded RNA structures and protein–RNA complexes, the three-point water models such as SPC/E, TIP3P and OPC3 might be more reasonable choices. However, we emphasize that our simulation set is not exhaustive. There will certainly be folded RNA systems (e.g., the 5s rRNA loop E tested here) for which the use of four-point water models will not pose any difficulties. Therefore, as a practical measure for future MD simulations employing four-point water models, we primarily recommend close monitoring of native tertiary interactions. Should any issues arise, they could, for example, be addressed using sHBfix, gHBfix (as demonstrated in this work), or mild restraints. It is not necessary to switch outright to three-point water models to mitigate these effects.

## Supplementary Material



## Data Availability

All MD trajectories and input files required to reproduce the simulations have been deposited at Zenodo (accession number 17854283).

## Notes

Additional details of the REST2 protocol utilized in simulations of the hTTR. Definitions of the characteristic disruptions of the native RNA folds. Definition of the gHBfix correction factors for OPC simulations. Additional simulated systems. Supporting figures and tables (PDF)
