# Supplementary material for: Destabilization of Structured RNAs by OPC and TIP4PD Water Models
Source: J Chem Theory Comput. 2026 Feb 10;22(4):2013–27. doi: 10.1021/acs.jctc.5c01678 (PMC12937104; doi:10.1021/acs.jctc.5c01678)
Supplement: Supplementary file 1 [file ct5c01678_si_001.pdf]

Destabilization of Structured RNAs by OPC and TIP4PD Water Models

Miroslav Krepl[1,*], Vojtěch Mlýnský[1], Agnesa Rusnáková[1], Pavel Banáš[2], Michal Otyepka[2,3], and Jiří Šponer[1]

[1]Institute of Biophysics of the Czech Academy of Sciences, Královopolská 135, 612 00 Brno, Czech Republic

[2]Czech Advanced Technology and Research Institute, CATRIN, Palacký University, Křížkovského 511/8, Olomouc 779 00, Czech Republic

[3]IT4Innovations, VSB-Technical University of Ostrava, 17. listopadu 2172/15, 708 00 Ostrava-Poruba, Czech Republic

*Corresponding author: Miroslav Krepl
E-mail: krepl@ibp.cz

Supporting Information Text

Additional comments on the definition of the hot zone region and simulation protocol in the REST2 simulations of the hTTR system. The two base pairs of the tetraloop receptor (A6:U36 and G8:C35) we have chosen to be included in the list of hot zone (scaled) atoms form two distinct types of H-bonds: (**a**) the standard base pairing H-bonds and (**b**) tertiary H-bonds with the GAAA tetraloop which constitute the TTR motif (Figure S2). Note that in this REST2 setup, (**a**) are directly scaled by λ, while (**b**) are scaled as √λ because the GAAA tetraloop lies outside the hot zone. Since our goal was actually to only accelerate the sampling of (**b**), we applied additional stabilization using 2 kcal/mol sHbfix (structure-specific HBfix) potentials for every base pairing H-bond in (**a**) to compensate for their weakening. This effectively focused the sampling enhancement onto the tetraloop-tetraloop receptor (TTR) interactions (**b**) (Figure S2). Note that we deliberately avoided including the GAAA tetraloop in the hot zone as this would have shifted most of the sampling enhancement toward exploring conformational variations within the GAAA tetraloop rather than the tertiary interactions forming the TTR motif. Such variations were not the target of this study. In addition, the GAAA tetraloop is less straightforward to stabilize with sHbfix restraints, making the two tetraloop receptor base pairs a more effective choice to define the hot zone.

Observables defining the characteristic disruptions of the native RNA folds. The "Time of RNA disruption" reported in Table 1 of the main text reflects large-scale, visually apparent perturbations of the RNA fold readily identifiable through visual inspection of the trajectories. To improve statistical rigor and ensure reproducibility, we additionally define a set of quantitative observables for detecting the disruptions. Namely, disruption of the L1 stalk RNA fold is monitored by the distance between the geometric centers of the heavy atoms of the tetraloop (residues 2187–2192 and 2143–2148 in the *H. marismortui* and *T. thermophilus* structures, respectively) and its receptor (residues 2111/2118 and 2152/2159 in the *H.m.* and *T.t.* structures, respectively), with distances exceeding 20 Å (*H.m.*) or 15 Å (*T.t.*) indicating a disrupted state. Disruptions of the L1 stalk protein–RNA interface are detected by monitoring the solvent-accessible interface area (see the main text Methods), where values below 2500 $Å^2$ signal loss of the interface. For the miniTTR-6 and hTTR systems, disruptions are monitored by the distance between the centers of geometry of the tetraloop and tetraloop-receptor H-bonding heavy atoms (see below and Figure 3 in the main text). Note that in hTTR, both TTR motifs are evaluated independently in this manner. Distances greater than 12 Å correspond to disrupted TTR motifs. Local or partial disruptions of the TTR motifs can be monitored by tracking the evolution of H-bond distances defined in Figure 3 of the main text. In the miniTTR-6, these include A49(O2′)-C85(O2′), G9(O2′)-A49(N1), A48(O2′)-U86(O2′), A48(N3)-U86(O2′), A7(N1)-A47(N6), A7(N6)-A47(N1), and G10(O2′)-C50(O2′) H-bonds. In the hTTR, the relevant H-bonds defining the first TTR motif are C35(O2′)-A66(O2′), G8(O2′)-A66(N1), U36(O2′)-A65(O2′), A65(N3)-U36(O2′), A6(N1)-A64(N6), A6(N6)-A64(N1), and G9(O2′)-C67(O2′). For the second TTR motif, the H-bonds are A23(O2′)-C78(O2′), G51(O2′)-A23(N1), A22(O2′)-U79(O2′), A22(N3)-U79(O2′), A21(N1)-A49(N6), A21(N6)-A49(N1), and C24(O2′)-G52(O2′).

Definition of the gHBfix OPC correction factors in REST2 simulations of hTTR. To define the strength of the gHBfix potentials required to stabilize the hTTR simulations in OPC, we summed the free-energy differences calculated for the OPC–SPC/E mixtures for every H-bond donor–acceptor combination listed in Table 3 of the main text. We stress that the values reported in main text Table 3 correspond to individual donor or acceptor groups. Therefore, to estimate the penalization of a specific H-bond in folded RNA systems, the values of both constituent groups (donor and acceptor) must be summed. In the current gHBfix implementation, the potentials are defined for *equivalent functional groups*. Thus, for example, the same gHBfix potential is applied to a base NH donor group – phosphate non-bonding oxygen interaction, regardless of the nucleotide to which the NH donor group belongs. Accordingly, all equivalent H-bond combinations derived from Table 3 for which only single gHBfix definition exists were averaged to obtain the corresponding gHBfix correction factors. The resulting values were then rounded up to the nearest tenth. This procedure yielded the following gHBfix coefficients:

```
-hb-NH-bO 0.3     # base nitrogen donor to bridging phosphate oxygen acceptor
-hb-NH-N 0.2      # base nitrogen donor to nitrogen acceptor
-hb-NH-nbO 0.2    # base nitrogen donor to non-bridging phosphate oxygen acceptor
-hb-NH-O 0.2      # base nitrogen donor to base oxygen acceptor
-hb-NH-OH 0.2     # base nitrogen donor to hydroxyl group oxygen acceptor
-hb-OH-bO 0.3     # hydroxyl group oxygen donor to bridging phosphate oxygen acceptor
-hb-OH-N 0.2      # hydroxyl group oxygen donor to base nitrogen acceptor
-hb-OH-nbO 0.2  # hydroxyl group oxygen donor to non-bridging phosphate oxygen acceptor
-hb-OH-O 0.3      # hydroxyl group oxygen donor to base oxygen acceptor
-hb-OH-OH 0.2     # hydroxyl group oxygen donor to hydroxyl group oxygen acceptor
```

For script implementing the gHBfix in AMBER based on this input, see Supporting Information of Ref.[1] or github (https://github.com/bussilab/ghbfix-training).

Additional simulated systems – Sarcin-Ricin Loop, the 5S rRNA Loop E, and the RNA three-way junction. The starting structures for the additional simulations (Table S1)**Error! Reference source not found.** were taken from the following PDB entries: ***3DW4*** (Sarcin–Ricin Loop; SRL),[2] ***354D*** (5S rRNA Loop E),[3] and ***2MTJ*** (RNA three-way junction).[4] Prior to system preparation, the overhanging unpaired terminal nucleotides in the SRL and Loop E structures were removed. The 2′-OH methylation present on a single nucleotide in the SRL was also removed, and the lone DNA nucleotide in Loop E was converted to RNA. For the RNA three-way junction, we used the first model of the NMR ensemble as the starting structure. All simulations of these three additional systems (see Figure S11 for their visualization) followed the protocol described in detail in the main text.

The SRL simulations showed some differences among the water models, with both OPC and TIP4PD struggling to maintain one of the important base–phosphate interactions (G16–U8 4BPh; see Figure S12). Although this interaction fluctuated in the SPC/E simulations as well, its population differed markedly: <10% in OPC and TIP4PD compared to

~48% in SPC/E. However, for the SRL simulations, the most important is to monitor the position of the G7 involved in the critical G7pU8 platform. The GpU platform is part of the larger "bulged-G" motif, where the G7 bulges out and forms a base triple with the reverse Hoogsteen A8:U17 base pair, interacting with other nucleotides to create a stable, compact structure.[2] The three H-bonds formed by G7 obviously fluctuated, when G7 occasionally and temporarily left the native conformation. Such events were always reversible in both the OL3 OPC and OL3 SPC/E simulations; that is, the G7 base eventually returned to its original position and re-formed its H-bonds. Thus, the native bound conformation of G7 was dominantly populated in the OL3 simulations. The stability of the G16–U8 4BPh interaction somewhat correlated with the G7 dynamics (Figure S12).

In contrast, the native position of G7 was irreversibly lost with the TIP4PD/DES-Amber combination in four simulations, consistently with earlier observations.[5] Curiously, the fifth TIP4PD/DES-Amber simulation behaved differently. Namely, in this trajectory, G7 remained in its original position and even the G16–U8 4BPh interaction was preserved. However, this replicate exhibited substantial disruptions elsewhere in the system not observed in any other simulation (Figure S12). This observation strongly suggests that the DES-Amber FF struggles to simultaneously maintain all the interactions characteristic for the SRL motif.

In contrast to the SRL motif, the simulations of the 5S rRNA Loop E revealed only minor differences between the water models and generally good performance overall. A small difference between the RNA FFs was observed whereas all simulations struggled to maintain the U(N3)–A(N7) H-bond of the two tWH U:G base pairs present (Figure S13). In this case the TIP4PD/DES-Amber combination reproduced this interaction somewhat better than OL3. However, the DES-Amber FF showed greater difficulty with maintaining several other interactions compared to the OL3-based simulations (Figure S13).

Lastly, we observed comparable performance of the OPC and SPC/E water models in the simulations of the RNA three-way junction. The OPC simulations exhibited occasional larger departures from the native structure (Figure S14), characterized by loss of multiple base pairs at the branching point of the junction. There were consequently also a slightly higher number of NOE distance violations with OPC than SPC/E (Table S4). However, we note that these differences were minor and largely reversible on the simulation timescale. We therefore suggest that, within the limits of our sampling, the performance of the SPC/E and OPC water models can be considered equivalent for this system.

Supplementary Information Figures

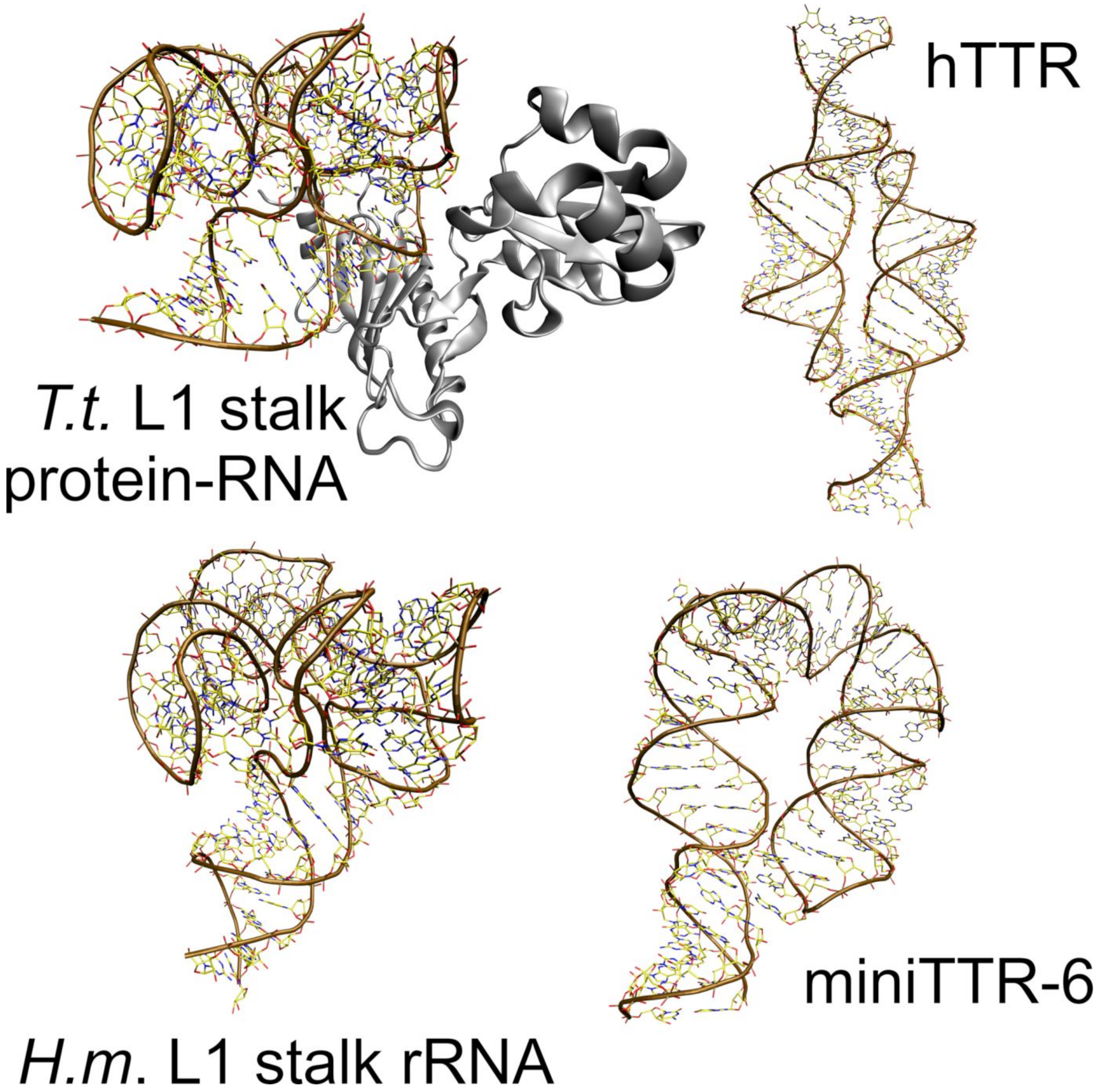

Figure S1. **Structured RNAs and the protein-RNA complex analyzed in the main text.** The T. thermophilus L1 stalk rRNA was also simulated in isolation. The RNA atoms are depicted as sticks with the backbone highlighted as a brown tube, while the protein is shown as grey ribbons.

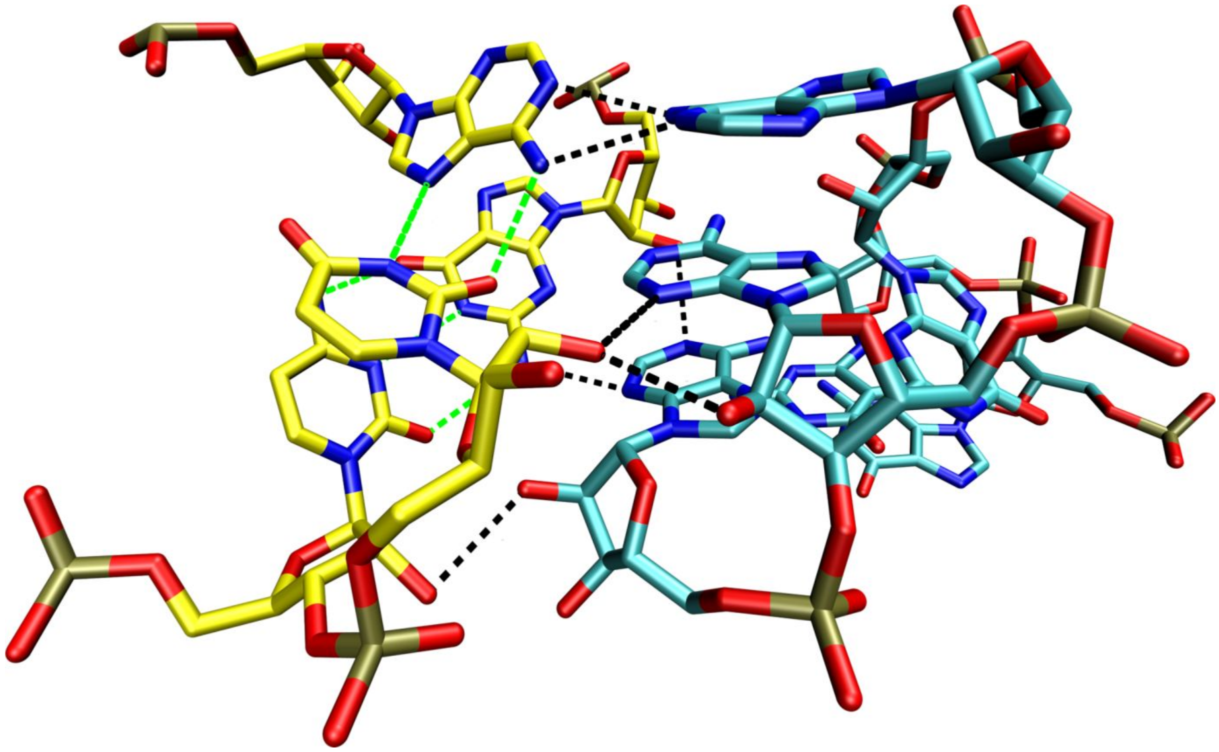

Figure S2. **Definition of the hot-zone region in REST2 enhanced-sampling simulations of the hTTR system.** The hot zone included only the *t*HW A6:U36 and *c*WW G8:C35 base pairs of the tetraloop receptor (carbons in yellow). To maintain their internal stability, these base pairs (base pairing H-bonds indicated by green dashed lines) were stabilized with a 2 kcal/mol sHBfix potential between the hydrogen and acceptor. This setup ensured that enhanced sampling targeted primarily the tertiary interactions with the GAAA tetraloop (carbons in cyan; H-bonds indicated by black dashed lines), without excessively perturbing the internal structures of either the tetraloop or the tetraloop receptor.

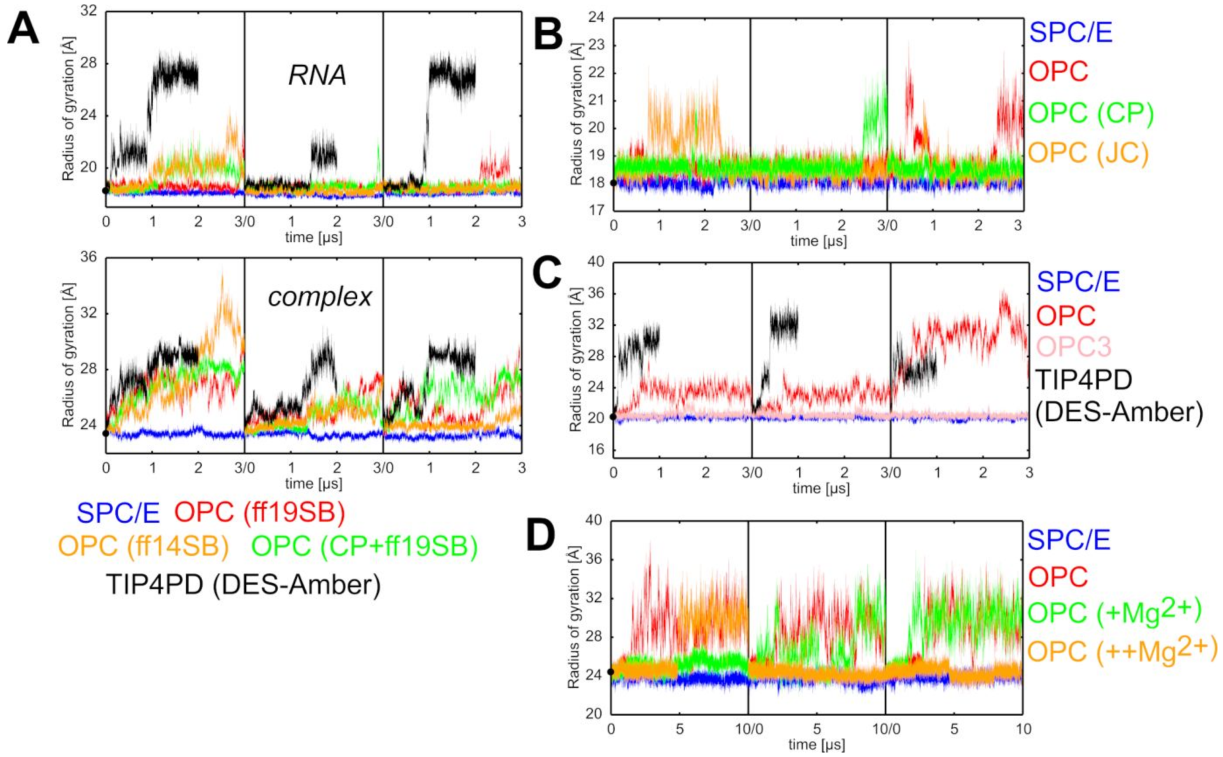

Figure S3. **Radius of gyration analysis of the simulated systems. A)** Time evolution of the average radius of gyration of the heavy atoms in the L1 stalk protein-RNA complex from *T.t.* Separate plots are shown for the RNA and the entire complex. **B)** Radius of gyration of the isolated L1 stalk rRNA from *T.t.* in individual simulations using selected water models. **C)** Same as B, but for the isolated L1 stalk rRNA from *H.m.* **D)** Radius of gyration of the RNA in selected simulations of the miniTTR-6 using different water models and ionic conditions (see the main text Methods and footnotes of the main text Table 1 for explanation of the abbreviations used to define the Mg^{2+} conditions). All the datasets are color-coded according to the neighboring legends. CP means that the $OL3_{CP}$ FF variant was used while JC means that the JC ions were applied (see main text). The experimental values of the radius of gyration are always indicated with black dots on the y-axes.

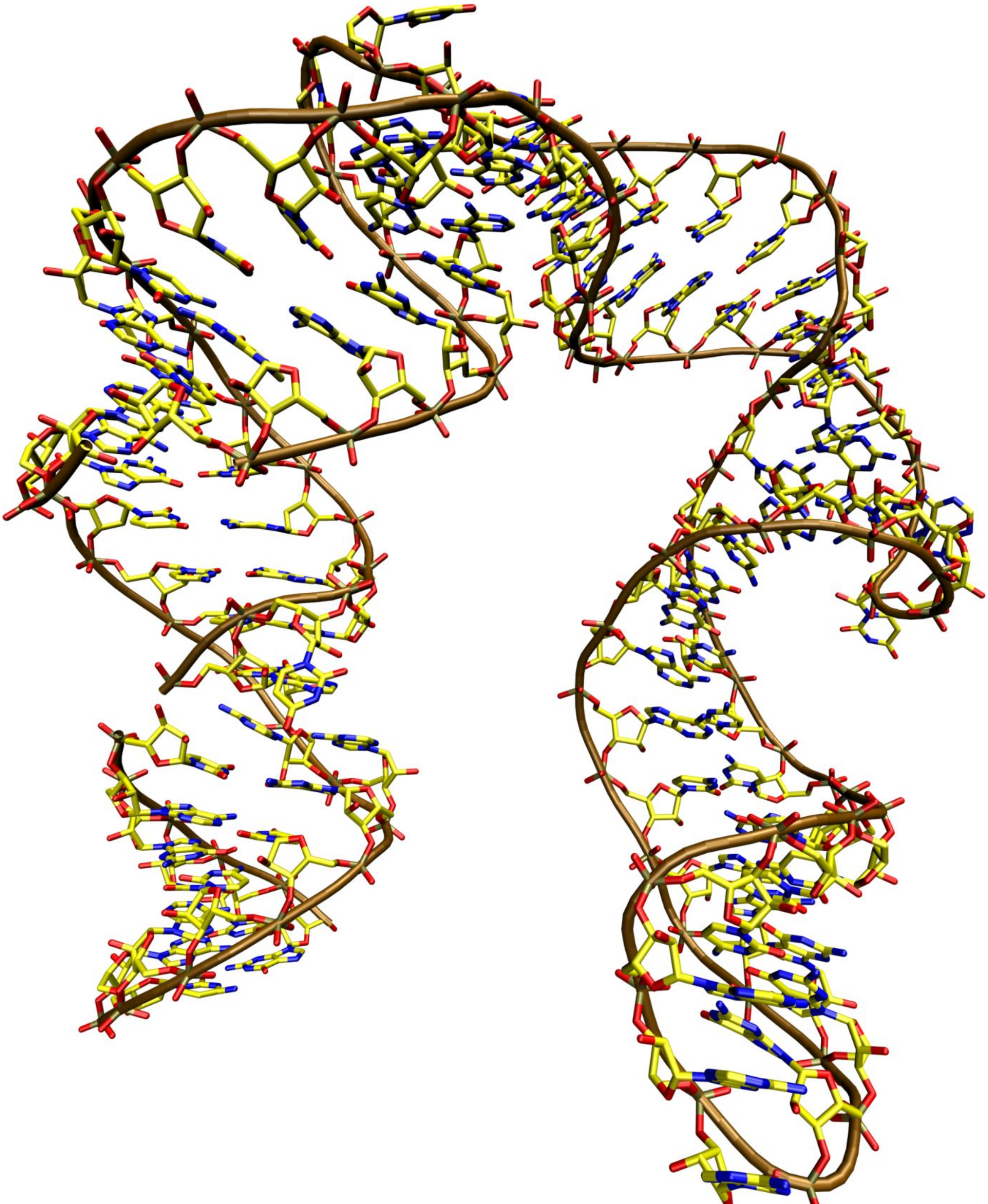

Figure S4. **Structure of the disrupted miniTTR-6 used as start for further MD simulations.** Compare with Figure S1. Shown is a snapshot from an OL3/OPC simulation, later used as the starting structure for additional OL3/SPCE and DES-Amber/TIP4PD simulations. In the OL3/SPCE simulations, the native RNA fold was eventually restored (see the main text and Figure S6).

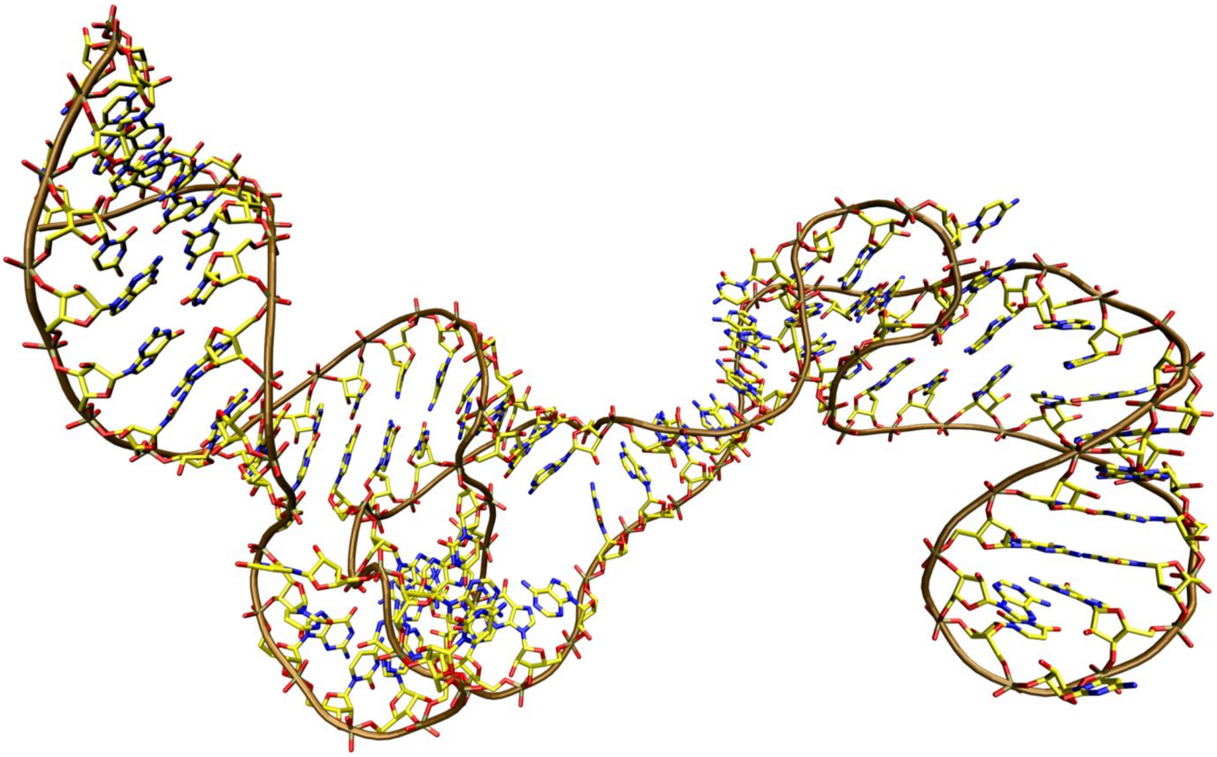

Figure S5. **Structure of the disrupted H.m. L1 stalk rRNA.** Compare with Figure S1. Shown is a snapshot from DES-Amber/TIP4PD simulations, in which the largest distortions were observed for this system. Many native non-canonical rRNA elements tended to become disrupted, giving way to an extended quasi-A-form structure, indicating an overall bias of the DES-Amber force field in favor of the A-form helix.

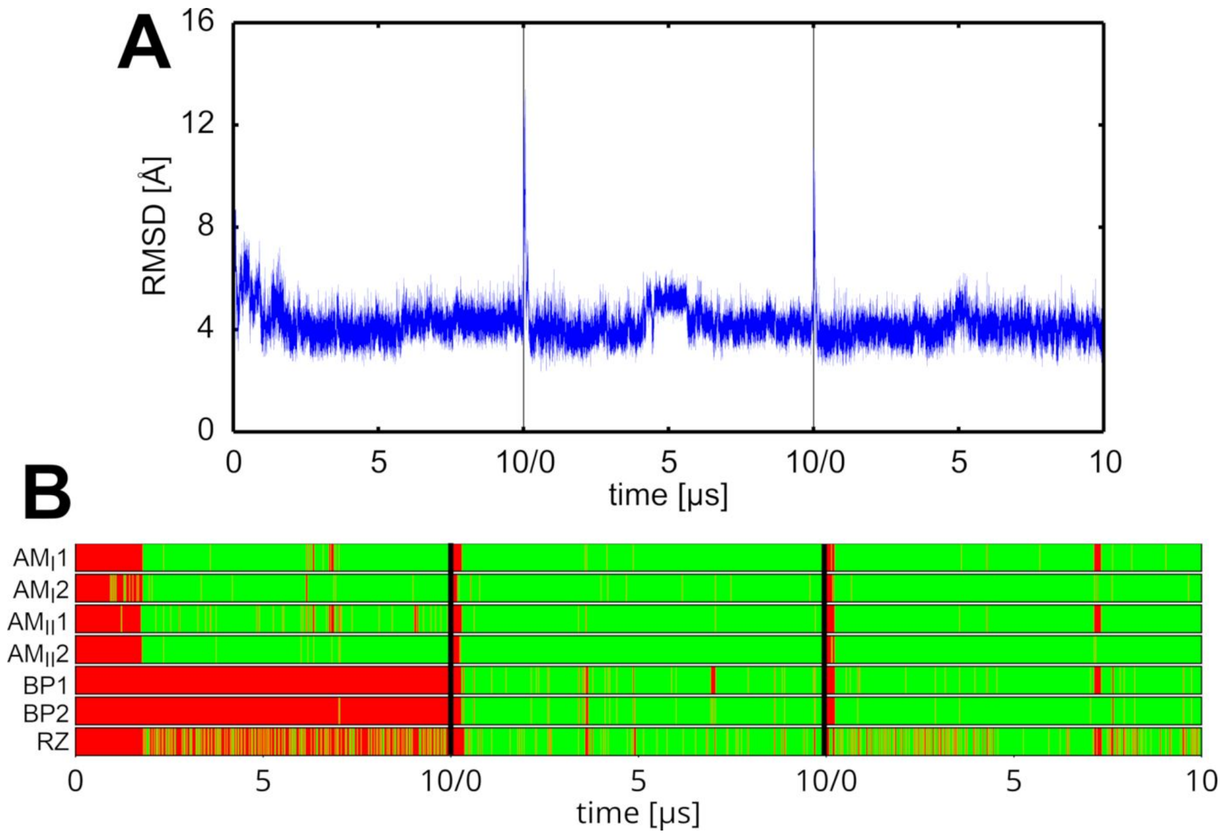

Figure S6. **Restoration of the native fold of miniTTR-6 in additional SPC/E simulations, started from a structure previously disrupted in OL3/OPC simulations. A)** Time evolution of the RNA RMSD in the restoration simulations. **B)** Time evolution of the signature TTR H-bonds. Green and red indicate presence and absence of the H-bond, respectively. A full restoration of the TTR was observed in two of the simulations and a partial restoration in one. See the main text Figure 3 for definition of the individual H-bonds and Figure S4 for the starting structure.

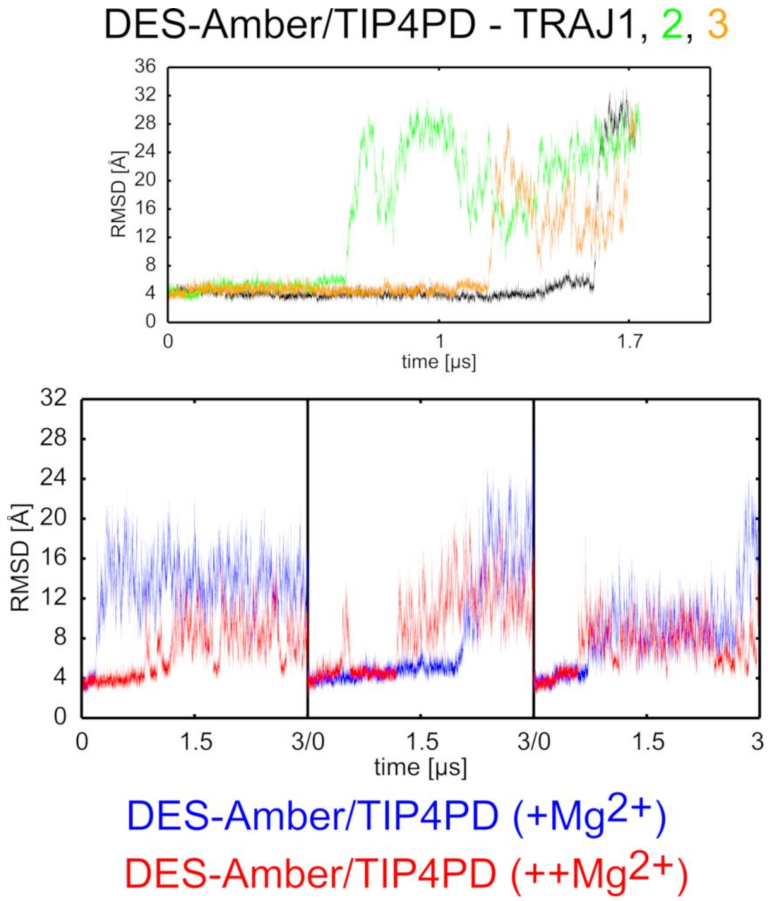

Figure S7. **Loss of the native RNA fold in simulations of miniTTR-6 using the DES-Amber FF and TIP4PD water model.** Time evolution of the RNA RMSD in simulations using different ionic conditions. Datasets are color-coded according to the legend.

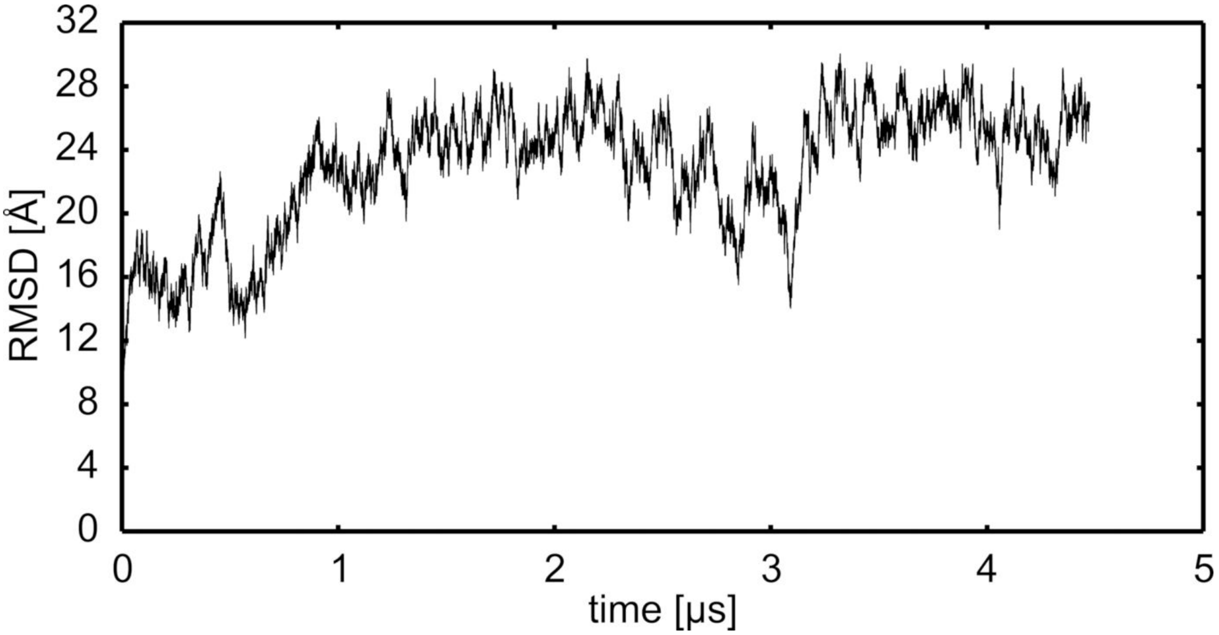

Figure S8. **Unsuccessful attempt to spontaneously restore the native fold of miniTTR-6 in DES-Amber/TIP4PD simulation.** The initial box size was substantially increased to prevent image clashes and potentially allow a spontaneous restoration. However, the system showed no tendency to return to the native fold and instead continued to deviate further from it. We subsequently terminated the simulation at ~4.4 μs.

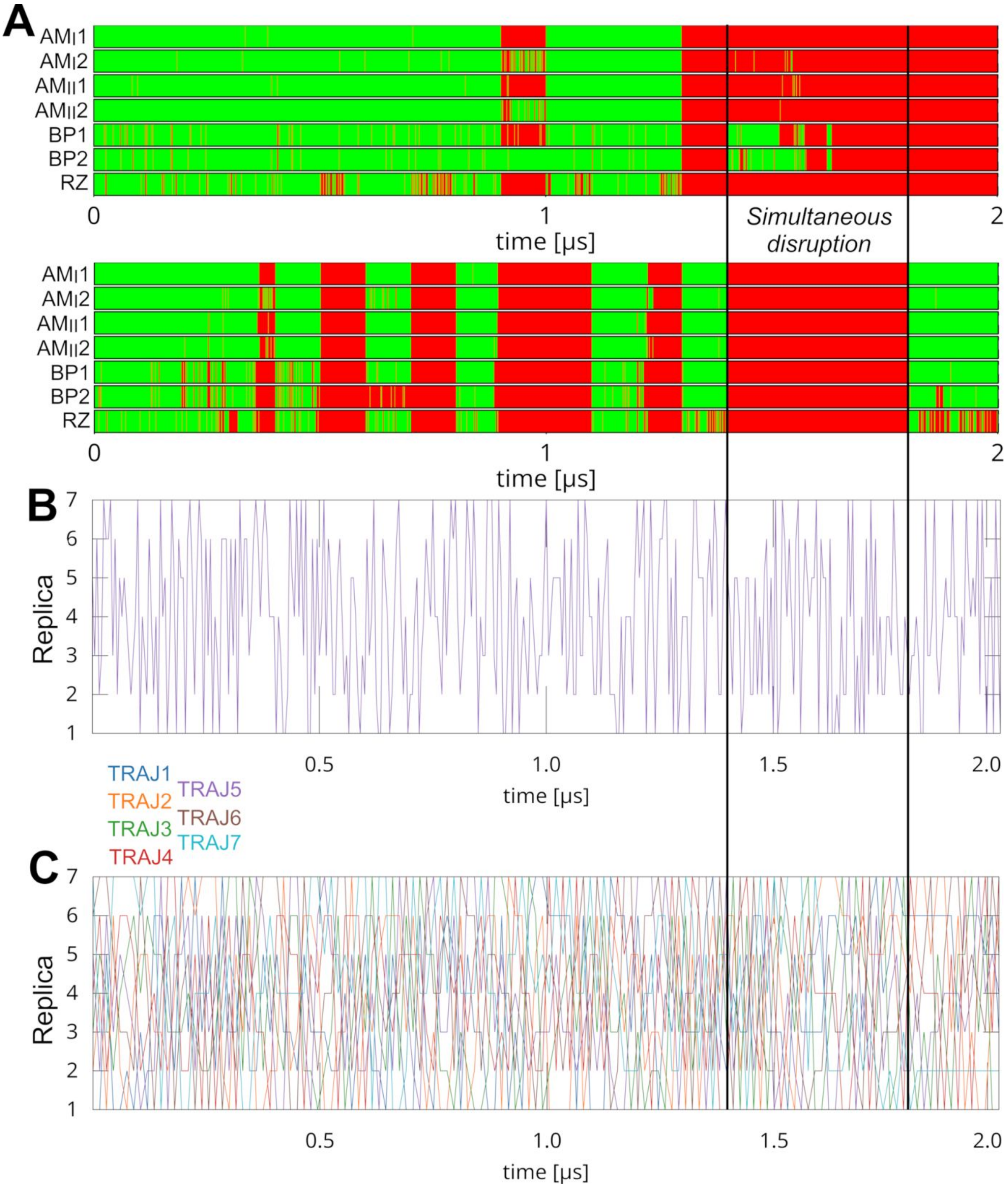

Figure S9. **Simultaneous disruption of the two TTRs in REST2 OL3/OPC simulations of hTTR. A)** Time evolution of the signature H-bonds of the TTR motifs in a demultiplexed continuous trajectory from the REST2 simulations with the OPC water model. Both TTRs are shown (top and bottom). The black vertical lines and the label highlight the instance of simultaneous disruption of both TTR motifs, followed by full spontaneous restoration of one. Green and red indicate presence and absence of individual H-bonds, respectively (see Figure 3 in the main text for definitions). **B)** Exchange pattern of the demultiplexed trajectory shown in panel A (TRAJ5) along the replica ladder. **C)** Same as panel B, but showing the exchange patterns for all the trajectories, color-coded according to the legend. For clarity, only every 2000th exchange is shown in the plots.

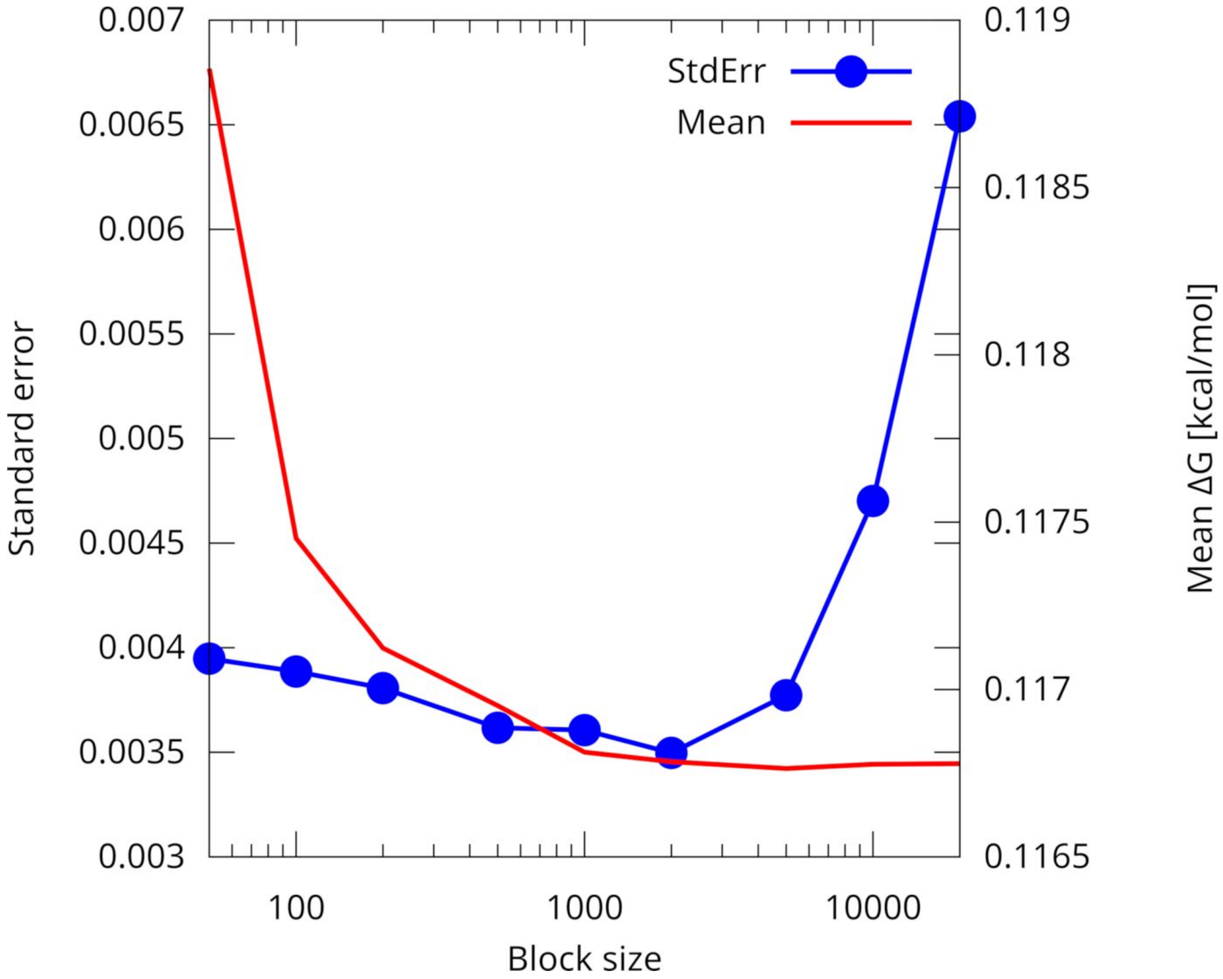

Figure S10. **Block-averaging convergence analysis of the simulations with mixed OPC–SPC/E water boxes.** Values of ΔG for water molecule binding to HO2′ group (main text Table 3) in one of the guanosine simulations are shown as a representative example. The mean value (solid red line) and its standard error (blue line with points) are shown as functions of block size. The total length of the simulation was 10 µs / 100 000 frames. The mean stabilizes for block sizes ≥ 2000 frames (≈200 ns), indicating that the estimate of the difference in water model affinity to the solute is well converged on the 10-µs-timescale.

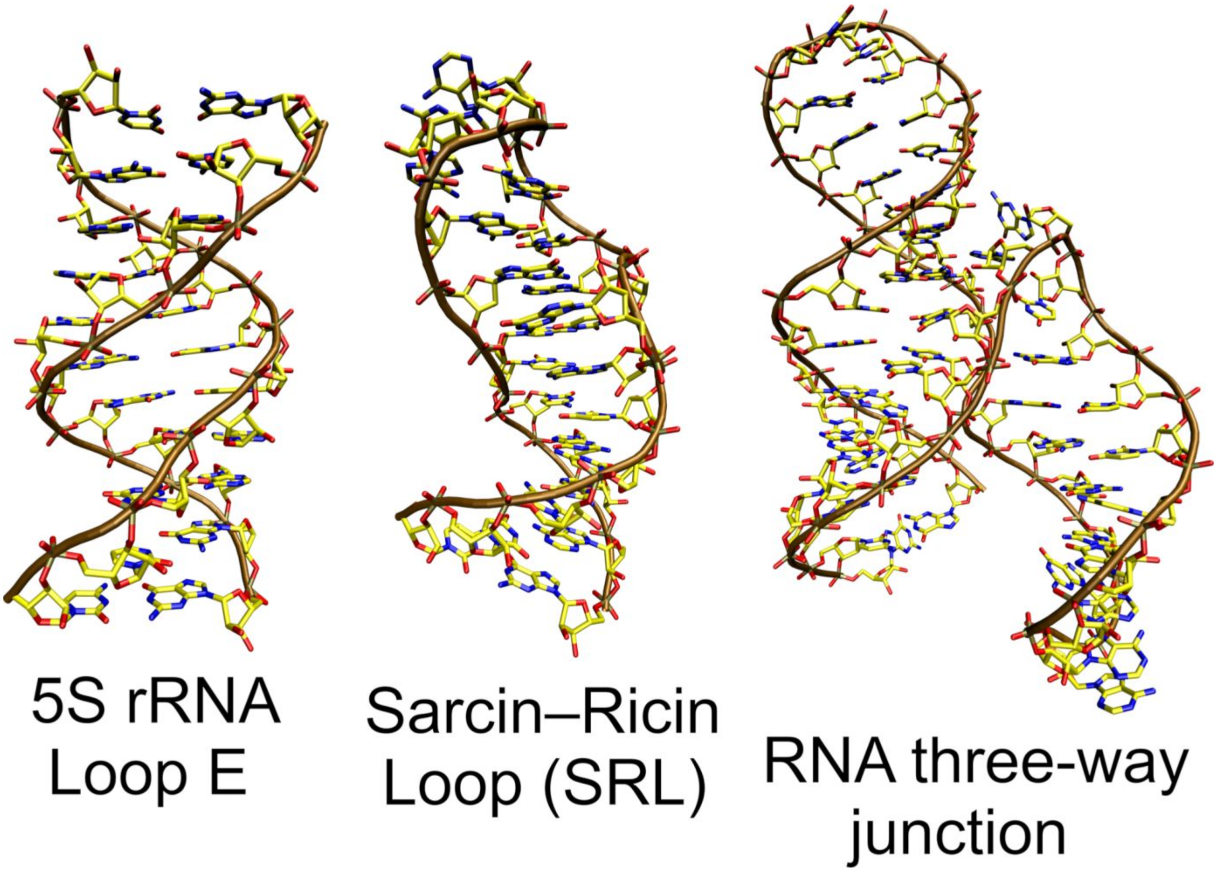

Figure S11. **Additional structured RNAs analyzed in the Supporting Information.** The RNA atoms are depicted as sticks with the backbone highlighted as a brown tube.

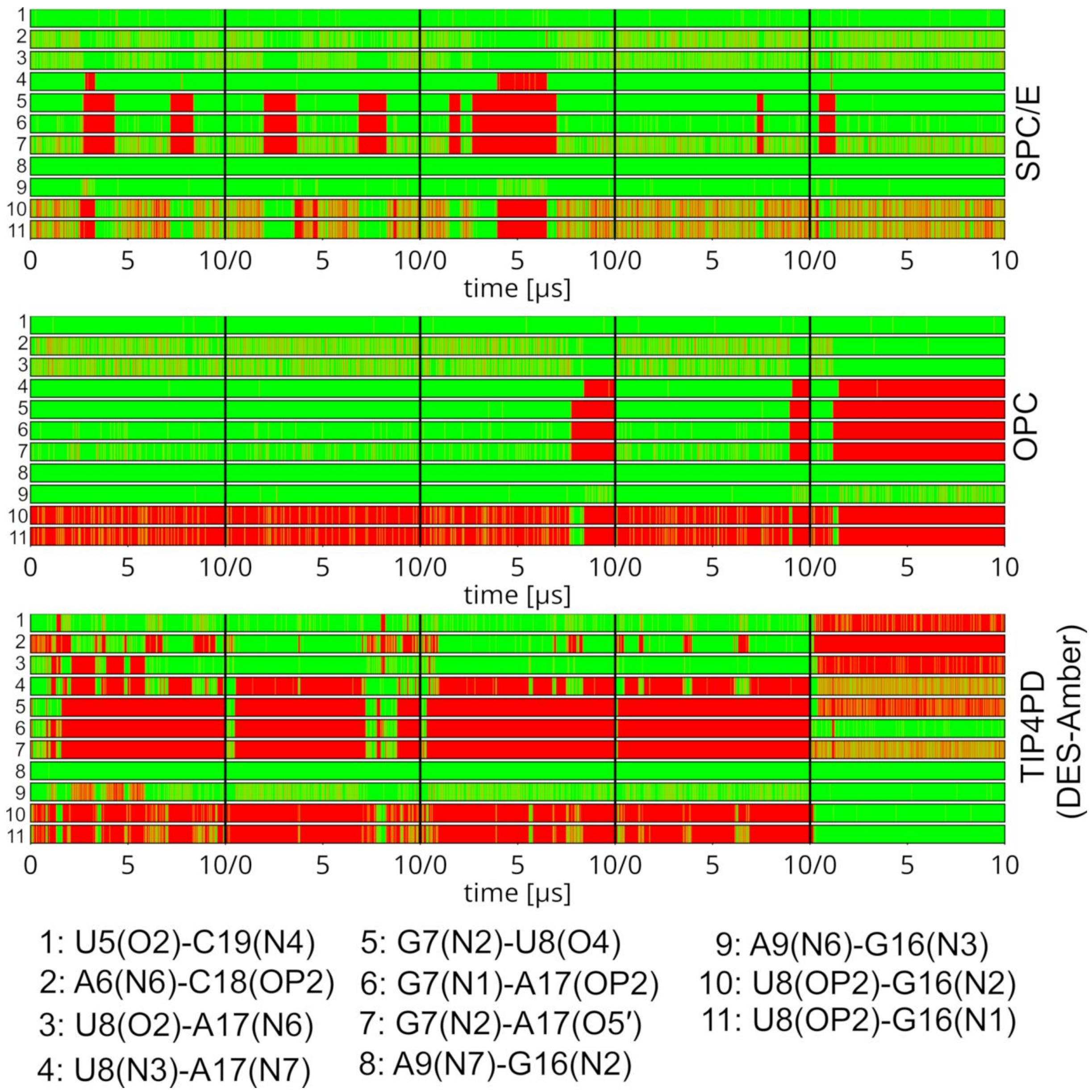

Figure S12. **MD simulations of the Sarcin–Ricin Loop (SRL) motif.** Time evolution of the native non-canonical H-bonds using different water models. Green and red indicate the presence and absence of each H-bond, respectively. The bar plots are numbered, and the corresponding H-bond interactions are listed in the legend below. Simulation residue numbering is used.

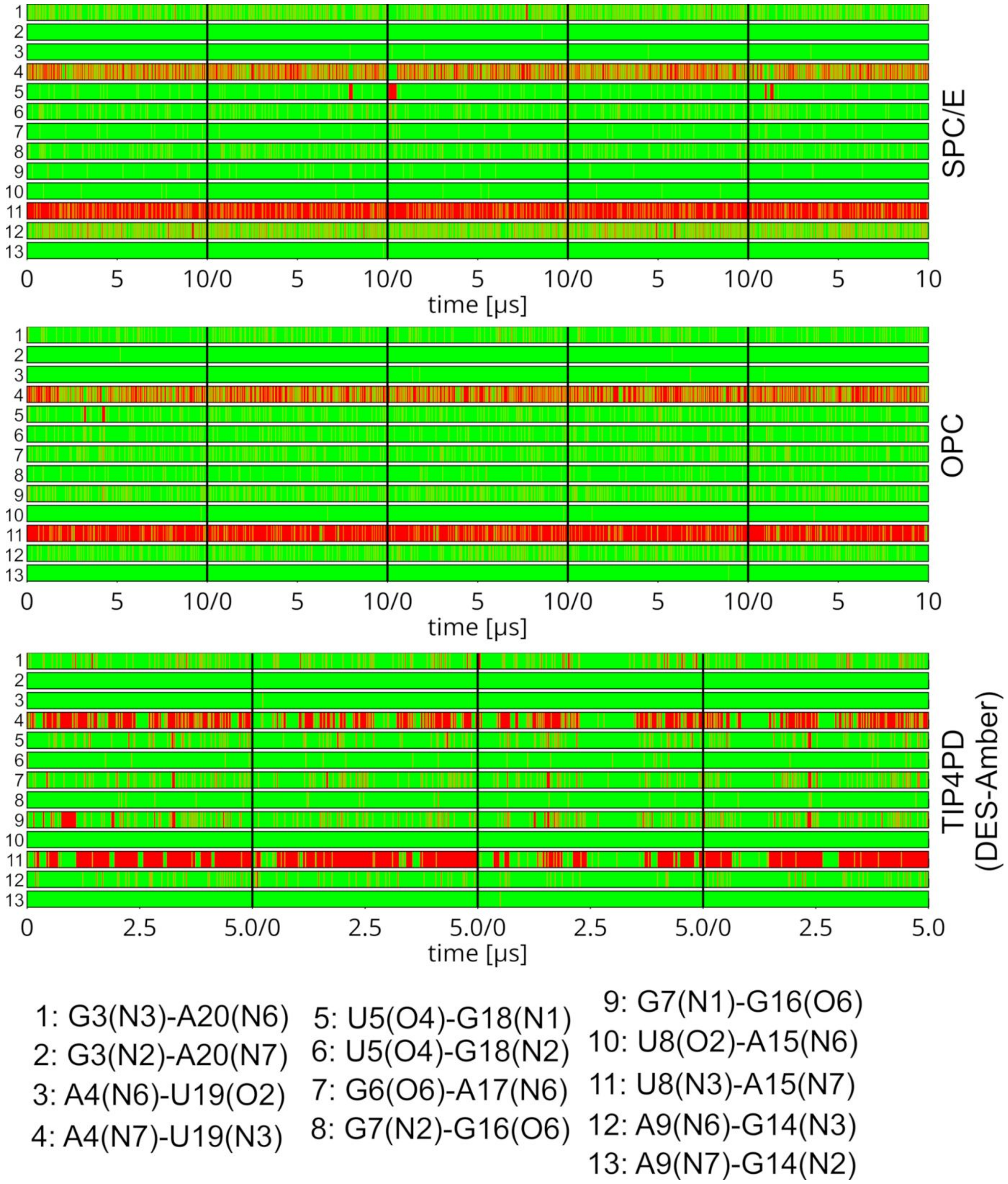

Figure S13. **MD simulations of the 5s rRNA Loop E.** Time evolution of the H-bonding interactions of the native non-canonical base pairs using different water models. Green and red indicate the presence and absence of each H-bond, respectively. The bar plots are numbered, and the corresponding H-bond interactions are listed in the legend below. Simulation residue numbering is used.

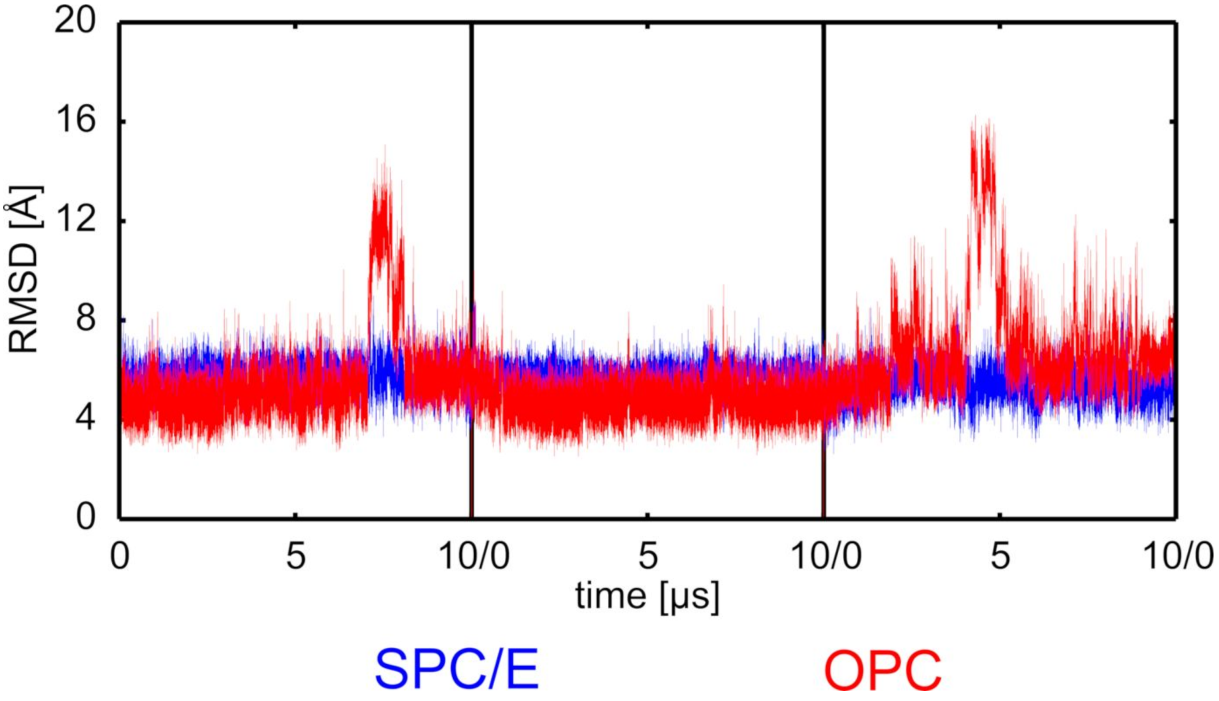

Figure S14. **RMSD time evolution in MD simulations of the RNA three-way junction.** Datasets are color-coded according to the legend below.

Supporting Information Tables

Table S1. List of additional MD simulations.

Force field	Water model	Ion parameters	Number of simulations × Length (μs)
Sarcin-Ricin loop (SRL)			
OL3	SPC/E	JC	5 × 10
$OL3_{CP}$	OPC	LM	5 × 10
DES-Amber	TIP4PD	CHARMM22	5 × 10
5s rRNA Loop E			
OL3	SPC/E	JC	5 × 10
$OL3_{CP}$	OPC	LM	5 × 10
DES-Amber	TIP4PD	CHARMM22	4 × 5
RNA three-way junction			
OL3	SPC/E	JC	5 × 10
$OL3_{CP}$	OPC	LM	5 × 10

Table S2. Statistics of intermolecular NOE distance violations in the REST2 MD simulations of the hTTR system.[a]

Force field–Water model	# NOE distances violated
**OL3–SPC/E**	3 of 30[b]
**OL3–OPC3**	3 of 30[b]
**OL3–OPC**	6 of 30
**OL3–TIP4PD**	10 of 30
**DESAMBER–TIP4PD**	12 of 30

[a]The experimental NOE violations in the REST2 simulations were calculated as the $(r^{-6})^{(-1/6)}$ weighted average of the NOE distances over the final 500 ns of the unscaled (basic) replica. These values were directly compared with the experimental upper-bound distances of the intermolecular NOE data. Individual NOE distances were considered violated in simulations when their ensemble-averaged values exceeded the corresponding experimental upper bound plus an error tolerance of 0.3 Å. Because the hTTR system consists of two identical monomers and two equivalent TTR motifs that cannot be experimentally distinguished in NMR spectra, the intermolecular NOE distances associated with symmetric proton pairs were concatenated prior to evaluating the NOE violations in simulations. This procedure resulted in a total of 30 intermolecular NOE distances being assessed.

[b]The three intermolecular NOE violations calculated for the SPC/E and OPC3 water models occurred consistently across all ensembles, irrespective of the water model used. We observed that these violations arise from minor geometric deviations within the GAAA tetraloops, which may stem from inaccuracies in the experimental data or from FF limitations in accurately describing tetraloop structures.[6]

Table S3. Difference in estimated binding free energy (in kcal/mol) between the specified water molecules and the specific H-bond donors and acceptors of the cytidine nucleoside.[a]

RNA atom	OPC3 – SPC/E	TIP4PD – SPC/E	TIP3P – SPC/E
**acceptors**			
**O5′**	0.01	0.03	0.05
**O2′**	0.02	0.03	0.10
**O3′**	0.01	0.03	0.05
**O2**	0.02	0.10	0.02
**N3**	0.03	0.07	0.06
**donors**			
**HO5′**	0.01	0.14	-0.06
**HO2′**	0.01	0.14	-0.03
**HO3′**	0.01	0.11	-0.05
**NH41**	-0.01	0.13	-0.01
**NH42**	-0.01	0.13	-0.05

[a]Negative number means the RNA donor/acceptor interacts more strongly with the SPC/E water. Values represent a combined simulation ensemble of all three simulations. A nucleoside was used for the simulations to avoid the need to include a water model-specific counter-ion.

Table S4. Statistics of NOE distance violations in the standard MD simulations of the RNA three-way junction.[a]

Force field–Water model	# NOE distances violated
**OL3–SPC/E**	46 of 1780
**OL3–OPC**	54 of 1780

[a]The experimental NOE violations were calculated as the $(r^{-6})^{(-1/6)}$ weighted average of the NOE distances over the final 2500 ns (the last 25% of frames) of each trajectory, with the replicates combined into a single ensemble. These values were directly compared with the experimental upper-bound distances of the intermolecular NOE data. Individual NOE distances were considered violated in simulations when their ensemble-averaged values exceeded the corresponding experimental upper bound plus an error tolerance of 0.3 Å.

Supporting Information References

(1) Kuhrova, P.; Mlynsky, V.; Zgarbova, M.; Krepl, M.; Bussi, G.; Best, R. B.; Otyepka, M.; Sponer, J.; Banas, P., Improving the Performance of the RNA Amber Force Field by Tuning the Hydrogen-Bonding Interactions. *J. Chem. Theory Comput.* **2019**, *15*, 3288-3305.
(2) Olieric, V.; Rieder, U.; Lang, K.; Serganov, A.; Schulze-Briese, C.; Micura, R.; Dumas, P.; Ennifar, E., A Fast Selenium Derivatization Strategy for Crystallization and Phasing of RNA Structures. *RNA* **2009**, *15*, 707-715.
(3) Correll, C. C.; Freeborn, B.; Moore, P. B.; Steitz, T. A., Metals, Motifs, and Recognition in the Crystal Structure of a 5S rRNA Domain. *Cell* **1997**, *91*, 705-712.
(4) Bonneau, E.; Legault, P., Nuclear Magnetic Resonance Structure of the III–IV–V Three-Way Junction from the Varkud Satellite Ribozyme and Identification of Magnesium-Binding Sites Using Paramagnetic Relaxation Enhancement. *Biochemistry* **2014**, *53*, 6264-6275.
(5) Tucker, M. R.; Piana, S.; Tan, D.; LeVine, M. V.; Shaw, D. E., Development of Force Field Parameters for the Simulation of Single- and Double-Stranded DNA Molecules and DNA–Protein Complexes. *J. Phys. Chem. B* **2022**, *126*, 4442-4457.
(6) Mlýnský, V.; Janeček, M.; Kührová, P.; Fröhlking, T.; Otyepka, M.; Bussi, G.; Banáš, P.; Šponer, J., Toward Convergence in Folding Simulations of RNA Tetraloops: Comparison of Enhanced Sampling Techniques and Effects of Force Field Modifications. *J. Chem. Theory Comput.* **2022**, *18*, 2642-2656.
